# Estimating Limit Reference Points for Western Pacific Leatherback Turtles (*Dermochelys coriacea*) in the U.S. West Coast EEZ

**DOI:** 10.1371/journal.pone.0136452

**Published:** 2015-09-14

**Authors:** K. Alexandra Curtis, Jeffrey E. Moore, Scott R. Benson

**Affiliations:** 1 Ocean Associates, Inc., under contract to Southwest Fisheries Science Center, National Marine Fisheries Service, National Oceanic and Atmospheric Administration, La Jolla, CA, United States of America; 2 Marine Mammal and Turtle Division, Southwest Fisheries Science Center, National Marine Fisheries Service, National Oceanic and Atmospheric Administration, La Jolla, CA, United States of America; Deakin University, AUSTRALIA

## Abstract

Biological limit reference points (LRPs) for fisheries catch represent upper bounds that avoid undesirable population states. LRPs can support consistent management evaluation among species and regions, and can advance ecosystem-based fisheries management. For transboundary species, LRPs prorated by local abundance can inform local management decisions when international coordination is lacking. We estimated LRPs for western Pacific leatherbacks in the U.S. West Coast Exclusive Economic Zone (WCEEZ) using three approaches with different types of information on local abundance. For the current application, the best-informed LRP used a local abundance estimate derived from nest counts, vital rate information, satellite tag data, and fishery observer data, and was calculated with a Potential Biological Removal estimator. Management strategy evaluation was used to set tuning parameters of the LRP estimators to satisfy risk tolerances for falling below population thresholds, and to evaluate sensitivity of population outcomes to bias in key inputs. We estimated local LRPs consistent with three hypothetical management objectives: allowing the population to rebuild to its maximum net productivity level (4.7 turtles per five years), limiting delay of population rebuilding (0.8 turtles per five years), or only preventing further decline (7.7 turtles per five years). These LRPs pertain to all human-caused removals and represent the WCEEZ contribution to meeting population management objectives within a broader international cooperative framework. We present multi-year estimates, because at low LRP values, annual assessments are prone to substantial error that can lead to volatile and costly management without providing further conservation benefit. The novel approach and the performance criteria used here are not a direct expression of the “jeopardy” standard of the U.S. Endangered Species Act, but they provide useful assessment information and could help guide international management frameworks. Given the range of abundance data scenarios addressed, LRPs should be estimable for many other areas, populations, and taxa.

## Introduction

The California drift gillnet fishery (CDGN) catches a long list of non-target species, including marine mammals, sea turtles, sharks, and teleosts [1]. The internationally agreed Code of Conduct for Responsible Fisheries [2] calls for minimizing unused catch and catch of threatened, endangered, and protected species, but often such catch cannot be eliminated while maintaining economically viable fisheries. Biological limit reference points (LRPs) for human-caused removals, which correspond to population thresholds defined by conservation objectives (also known as minimum management objectives) for populations, support an ecological risk assessment approach to management of human impacts on marine wildlife and ecosystems, allowing strategic prioritization of conservation concerns [3–5]. Under the U.S. Marine Mammal Protection Act, incidental catch of marine mammals must remain below such an LRP: Potential Biological Removal (PBR) is analogous to a buffered estimate of Maximum Sustainable Yield for a fish stock, such that population depletion is avoided with high probability in the face of biological and management uncertainty [4,6]. Similar LRPs are needed for other taxa of conservation concern caught in the fishery.

**Fig 1:**
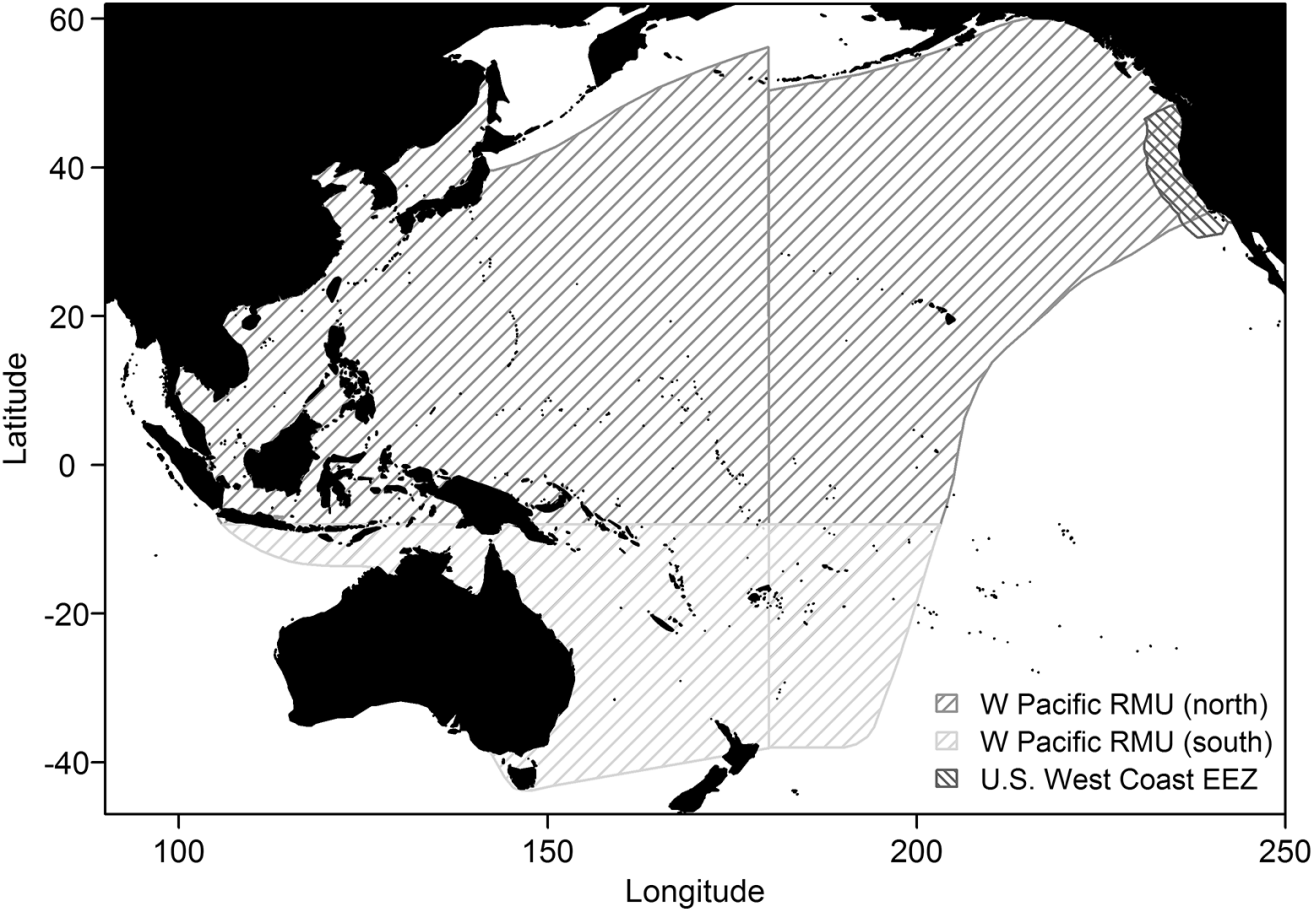
Map of study areas. Map of western Pacific leatherback Regional Management Unit [8,11,12] and U.S. West Coast EEZ [13], highlighting RMU area north of 8°S, which represents the range of boreal-summer-nesting western Pacific leatherback turtles for the purposes of this study. Figure created in R 3.1.0 [14] with the help of the rgdal [15], sp [16], rgeos [17], maps [18], maptools [19], and extrafont [20] packages, with coastline data from the CIA World DataBank II via the mapdata package [21].

One high-profile bycatch species in the CDGN is the leatherback turtle (*Dermochelys coriacea*), due to its Endangered status under the U.S. Endangered Species Act (ESA). Pacific leatherback populations are declining at an alarming rate [7], and the western Pacific leatherback regional management unit (RMU, Fig 1) [8], which includes the genetic stock interacting with the CDGN, is classified as Critically Endangered on the International Union for the Conservation of Nature (IUCN) Red List of Threatened Species [9]. Leatherback bycatch in the CDGN has declined since the 1990s due to the declining population, declining effort in the fishery, and implementation in 2001 of the Pacific Leatherback Conservation Area, a large seasonal area closure that encompasses much of the U.S. West Coast Exclusive Economic Zone (WCEEZ) off central California and Oregon [10]. However, disagreement over whether the area closure is sufficient or excessive continues to consume time and resources for numerous stakeholders and managers. An LRP for leatherbacks could help evaluate whether current removals of leatherbacks by the fishery are consistent with conservation objectives for the population. (NB: data and software used for Fig 1 are detailed in the caption [11–21].)

The California Current region serves as a seasonal foraging ground for large juvenile through adult leatherback stages, which show high interannual foraging site fidelity [22]. Adults from this region migrate to the western Pacific to breed [22], joining turtles from several other foraging areas to make up the western Pacific leatherback genetic stock, which nests in Indonesia, Papua New Guinea, Vanuatu, and Solomon Islands [23]. Nest counts at index beaches in West Papua, Indonesia, where the majority of nesting for the western Pacific genetic stock occurs, have been declining steadily for decades at an estimated 6% per year [24].

The decline of western Pacific leatherbacks is a complex problem. Their vast range and diversity of habitats expose them to many sources of human-caused mortality in multiple management jurisdictions across the Pacific Ocean [25,26]. Agreement and coordination among all responsible parties on limiting human-caused mortality of western Pacific leatherbacks is therefore highly unlikely in the near future. Instead, incremental progress may be made by managing local impacts based on local abundance, as is recommended for marine mammal stock assessments under the U.S. Marine Mammal Protection Act [5,27]. Using this approach, human-caused removals of western Pacific leatherbacks in the WCEEZ (Fig 1), where the CDGN occurs, would be managed based on their local abundance.

In the United States, *de facto* fishery-specific limits for sea turtle removals are already in place for many fisheries in the form of take exemptions resulting from Biological Opinions issued by the National Marine Fisheries Service pursuant to ESA Section 7 consultation requirements. Biological Opinions are an evaluation of whether the anticipated level of incidental catch in a fishery is expected to jeopardize the species, i.e., reduce appreciably its probability of survival and recovery (16 U.S Code Section 1531). The level of anticipated removals evaluated in a Biological Opinion does not reflect the maximum level of removals that could occur before jeopardy would be expected, so exempted take levels do not correspond to biological reference points. Moreover, the incremental impact of any one fishery or other activity may not be likely to trigger a jeopardy finding, particularly given typically high biological uncertainty for sea turtles, even though the cumulative impact across all fisheries may be detrimental [28]. An LRP-based approach like PBR could inform jeopardy determinations to support integrated management of cumulative impacts across activities and consistent management across regions and populations.

A primary objective of this study was to estimate a local LRP for human-caused removals of western Pacific leatherbacks in the WCEEZ to help inform management of bycatch in the CDGN. A broader objective was to demonstrate an approach that can be applied to a wide array of taxa, within different national or international frameworks, to support bycatch management under an ecosystem approach. To demonstrate the adaptability of reference point estimation across a wide spectrum of data availability for local abundance, we took three approaches, with increasing levels of information on local abundance, to estimating a local LRP. We largely followed the procedure and recommendations outlined by Curtis et al. [5] for estimating LRPs for non-teleost marine vertebrates, such as marine turtles. First, the population unit, population thresholds, and risk tolerance for each threshold were specified. An appropriate LRP estimator was identified for each of the three approaches. The thresholds and associated risk tolerances then served as performance criteria in a management strategy evaluation (MSE) for each approach to tune the corresponding LRP estimator. Sensitivity trials explored the effects of bias in key parameters of the population model underlying the LRP estimators and their evaluation. The resulting LRPs provide a basis for evaluating human-caused mortality of western Pacific leatherback turtles in the WCEEZ.

## Methods

### Step 1: Specify conservation objectives

Estimating LRPs first requires that general conservation objectives for a population, such as maintaining productivity, be refined to specific objectives in the form of biological thresholds, above which the population should be maintained or to which it should be allowed to rebuild. This step also requires delimiting the population unit to which the conservation objectives apply, and setting risk tolerances and time horizons that can be combined with the population thresholds to evaluate LRP performance through simulation. To specify conservation objectives for this analysis, we followed guidelines based on international agreements, precedent, and best practice [5]. These objectives are not direct expressions of legal species conservation standards under the ESA, such as the “jeopardy” standard, and would need to be revisited in the event of management application of LRPs within that context.

#### Step 1A: Define population unit of interest

The majority of nesting activity of western Pacific leatherbacks occurs on the beaches of West Papua, Indonesia, with lower levels of nesting in Papua New Guinea, Solomon Islands, and Vanuatu [9,23]. Two distinct annual nesting peaks occur, in boreal summer and winter [29]. Telemetry data show that females nesting in boreal summer exhibit strong fidelity to their respective foraging grounds in the North Pacific Ocean (including the California Current region) and South China Sea [22,30], while boreal winter nesters migrate south to forage [22]. A drift simulation study suggests that seasonal currents may maintain the observed separation by carrying hatchlings from these two nesting peaks in different directions [31]. Given the evidence for at least partial demographic independence, and the importance of managing based on demographically independent units [32,33], the population unit defined for this study was boreal-summer-nesting western Pacific leatherback turtles (henceforth “boreal summer nesters”, referring to the population inclusive of males and non-adults). We assumed the population to be closed, although some exchange between boreal summer nesters and boreal winter nesters may occur, particularly in the months between peak nesting seasons.

Jamursba Medi and Wermon beaches (JMW) on Bird’s Head peninsula in West Papua constitute roughly 75% of nesting for western Pacific leatherbacks as a whole, and a still greater (though unquantified) majority of boreal summer nesting [23]. Time series of nest counts at JMW provide the best available information on abundance of boreal summer nesters [24], so we used these as the basis for estimating total abundance for boreal summer nesters. This represents a minimum estimate, since it omits any turtles nesting at unmonitored beaches in the region.

#### Step 1B: Establish population thresholds

We considered two conservation objectives: maintaining productivity and avoiding precipitously low population sizes. We specified the first–and primary–objective as a population threshold at the maximum net productivity level (*N*_MNP_; analogous to the maximum sustainable yield level used in fisheries assessments), in keeping with previous work and best practices in limit reference points [5,6,34,35]. We specified the second objective as a safeguard threshold of 10% of virgin population size (*N*_collapse_ [36]) to ensure that even where uncertainty is large, extremely undesirable population outcomes are avoided with high probability[5]. Current nest counts at JMW are roughly one tenth of the highest historic counts [24], so in this case the *N*_collapse_ threshold also corresponds to an objective of preventing further population decline, and thus corresponds well to a recovery criterion in the U.S. recovery plan for Pacific leatherback populations [37]: “Nesting populations at ‘source beaches’ are either stable or increasing over a 25 year monitoring period.” The other population-level-oriented criterion in the U.S. recovery plan stipulates: “Each stock must average 5,000 (or a biologically reasonable estimate based on the goal of maintaining a stable population in perpetuity) [females estimated nesting annually] over six years”, which probably sets a higher bar than the *N*_MNP_ threshold, given that the maximum recorded estimate of females nesting annually at JMW was around 3000 in 1984, including both boreal summer and winter [24].

We also explored a third, rebuilding-oriented objective: to limit the time required by a population to rebuild to the *N*_MNP_ threshold to no more than 10% longer than the time it would take without human-caused mortality. This objective was used to tune the PBR estimator for depleted populations under the MMPA [6]. The combination of objectives used to tune the PBR estimator under the MMPA (this expedited-rebuilding objective and the *N*_MNP_ objective) “shares the general intent of the jeopardy standard of the ESA in terms of looking at both the continued existence and recovery of a population” [38]. The ESA does not explicitly equate a delay to an impact on likelihood of recovery, but has been applied in that sense for other species [28,39,40].

#### Step 1C: Establish risk tolerances and time horizons

Risk tolerances for failing to meet specified conservation objectives may depend on the objective and on current population status. For example, acceptable risk of falling below *N*_collapse_ may be less than that of falling below *N*_MNP_, and risk tolerance may be lower overall for endangered than non-threatened populations [5]. An assessment of current status in terms of IUCN Red List Criterion E for boreal summer nesters, based on the time series at JMW, aligned with a classification of Critically Endangered (S1 Text), in agreement with the published assessment for the western Pacific leatherback RMU as a whole [9]. We used suggested risk tolerances for populations classified as Endangered or Critically Endangered on the IUCN Red List of 5% for falling below *N*_MNP_ and 2.5% for falling below *N*_collapse_, evaluated after two generations [5]. We did not specify a risk tolerance for the expedited rebuilding objective.

### Step 2: Choose a limit reference point estimator

We used three approaches to estimating LRPs for leatherback turtles in the WCEEZ based on different types of information on local abundance, each with different considerations for LRP estimator choice. We first review the estimators used, then the three approaches to LRP estimation with choice of estimator for each.

#### The PBR and RVLL estimators

Two LRP estimators were used: the PBR estimator and the Reproductive Value Loss Limit (RVLL) estimator [35]. The PBR estimator assumes that removals of all life stages have equal impact on the population, or that impacts are not age- or stage-selective. These assumptions rarely hold for sea turtles; an increase in reproductive value by as much as two orders of magnitude from egg to adult [41,42] combined with ontogenetic migration among areas and habitats ensures that individual human activities are age- and size-selective, with widely varying consequences for the population given the same mortality [42]. To address this problem, Curtis and Moore [35] generalized the PBR estimator to an age-structured context. The resulting RVLL estimator expresses biological reference points in terms of adult equivalents (i.e., reproductive value relative to adults [43]) rather than individuals, thereby standardizing removals of different life stages to a common currency [44,45].

The published form of the PBR estimator is

$$\text{PBR}=\;0.5\;R_{\text{max}}\;N_{\text{min}}\;F_{\text{r}}$$ (1)

where *R*_max_ is maximum annual net population growth rate; 0.5 is the fraction of *R*_max_ corresponding to *N*_MNP_ under logistic growth; *N*_min_ is a minimum (20^th^ percentile) abundance estimate of the population that ensures that the risk of falling below *N*_MNP_ is within a specified tolerance; and *F*_r_ is a factor (restricted under the MMPA to be between 0.1 and 1) that accounts for potential biases in inputs and in the underlying population model, as well as for other management considerations [6]. The RVLL estimator proposed by Curtis and Moore [35] substitutes *R*_max_ with ${\hat{\lambda }}_{m}-1$, where ${\hat{\lambda }}_{m}$ is the estimated eigenvalue of the population transition matrix at very small population sizes, and *N*_min_ with ${\hat{N\prime }}_{\text{min}}$, the minimum abundance estimate in terms of adult equivalents.

We modified the usage and notation of the PBR and RVLL estimators for this study. First, we carried over the use of ${\hat{\lambda }}_{m}-1$ in place of *R*_max_ to the PBR equation to apply it in an age-structured context. Second, we replaced *F*_r_ (and the corresponding uncertainty factor in the RVLL equation) with *f*_a_, an adjustment factor, whose label better captures its range of purposes. The value for *f*_a_ was determined based on MSE (detailed in Step 5). Finally, rather than identifying a percentile of the abundance distribution as *N*_min_ or ${\hat{N\prime }}_{\text{min}}$, we used MSE to identify a percentile of the probability distribution for the LRP that satisfied performance criteria. This allowed us to account for uncertainty in ${\hat{\lambda }}_{m}$ as well as abundance. A distribution for the LRP was generated by drawing 2000 random samples from the probability distributions for ${\hat{\lambda }}_{m}$ and *N* or *N'*. The final equations used for PBR and RVLL were

$$\text{PBR}=\;{\left[0.5\;\left({\hat{\lambda }}_{m}-1\right)\;\hat{N}\;f_{\text{a}}\right]}_{\text{min}}$$ (2)

$$\text{RVLL}=\;{\left[0.5\;\left({\hat{\lambda }}_{m}-1\right)\;\hat{N\prime }\;f_{\text{a}}\right]}_{\text{min}}$$ (3)

#### Three approaches to local LRP estimation

The three approaches used for local LRP estimation for leatherbacks in the WCEEZ are summarized in Table 1, with more detail on abundance estimation provided in Step 4. The choice of LRP estimator for each approach followed guidance for choosing fisheries-independent LRP estimators provided in Curtis et al. [5] and is summarized below. The “naïve” and “survey” approaches illustrate potential ways forward for different situations of data availability. The “tag” approach provides the most valid inference for the current case study, because it used the least biased information and allowed for the most complete accounting of uncertainty.

**Table 1:** Three approaches to limit reference point calculation.

Approach	Information on Local Abundance Relative to Total	LRP Estimator
Naïve	None; assume proportion of total population in WCEEZ equals the proportion of total range within the WCEEZ	RVLL
Survey	Near-shore aerial surveys of abundance	PBR
Tag	Proportion of satellite-tagged boreal-summer-nesting females at JMW migrating to WCEEZ, size composition in drift gillnet bycatch data, and size distribution of nesting adults	PBR

Three approaches used to calculate local limit reference points (LRPs) for western Pacific leatherback turtles in the U.S. West Coast Exclusive Economic Zone (WCEEZ). JMW = Jamursba Medi and Wermon beaches in West Papua, Indonesia, where the vast majority of nesting for boreal-summer-nesting western Pacific leatherbacks occurs. RVLL = Reproductive Value Loss Limit. PBR = Potential Biological Removal.

The “naïve” approach provides an option for local LRP estimation in the absence of information about local abundance and life stages. It exploits the null hypothesis that a population’s abundance is distributed evenly across its range, so local abundance is estimated from the fraction of that range contained in the local jurisdiction, multiplied by an estimate of total population size [5]. To allow for potential bias in age classes in the WCEEZ relative to the total population, which would affect population impact of local human-caused removals, we used the RVLL estimator to calculate this LRP in terms of adult equivalents.

The “survey” approach estimated abundance from local surveys, which can provide the most direct estimates of local abundance if they are comprehensive. However, the aerial surveys off California used for this approach were restricted to the nearshore [46], so they provide a minimum estimate. The estimate was not extrapolated to the WCEEZ, because the surveys focused on prime foraging habitat and were thus not representative of mean density in the WCEEZ. Based on the assumption that the CDGN is not selective with respect to available size classes in the WCEEZ and that any existing selectivity is of limited consequence due to the limited range of reproductive values represented in the WCEEZ [22,47], and given a direct estimate of abundance, we chose the PBR estimator to calculate this LRP in terms of individuals.

The “tag” approach estimated local abundance relative to total nesting females based on the proportion of satellite-tagged nesting females migrating to the WCEEZ to forage [22], sex ratio observed in the WCEEZ [22], size composition of leatherback bycatch in the CDGN [48], and size composition of boreal-summer-nesting females observed at JMW [22]. Based on the assumed lack of and inconsequence of fishery selectivity, and the location-specific estimate of abundance for all stages, we used the PBR estimator for this approach.

### Step 3: Estimate maximum productivity ${\hat{\lambda }}_{m}$

The only published estimate for maximum potential population growth rate for western Pacific leatherbacks is based on severely limited information about leatherback catch and fishing effort through time [49]. We therefore estimated maximum productivity ${\hat{\lambda }}_{m}$ based on observed values for leatherback populations elsewhere that are recovering from very low abundance. The annual rate of increase in nesting female leatherbacks in South Africa is estimated to be 4 to 5.6% [50,51]. Mean estimates for the annual increase in female leatherbacks nesting in St Croix (U.S. Virgin Islands) and those nesting in Florida range from 9 to 13% [52,51,50,53]. Western Pacific leatherback turtles have a similar remigration interval, clutch size, and clutch frequency to those observed in St. Croix [9,24,51,54], where the highest population productivity was observed. Thus, to the extent that reproductive output can be considered an index of population productivity [55], treating these populations as proxies in estimating potential productivity of western Pacific leatherbacks is a reasonable approach. However, in St. Croix, population recovery has been boosted by a long-term nest protection program [52]; such programs have not achieved the same level of success for western Pacific leatherbacks. When adjusted to account for the difference in hatchling production between nesters at St. Croix and West Papua (S2 Text), the upper mean estimate of population growth rate is 6%. We parameterized population growth rate as ${\hat{\lambda }}_{m}$ ~ U(1.04,1.06), i.e., as a uniform distribution on the interval [1.04,1.06].

### Step 4: Estimate local abundance

The mean number of females nesting annually for the boreal-summer-nesting stock in 2014, ${\hat{n}}_{2014}$, was estimated from population forecasts from the extinction risk assessment for the population (S1 Text). Three approaches summarized in Step 2 and detailed below were used to derive local (WCEEZ) estimates from these population-level values.

#### “Naïve” approach

We estimated local abundance in terms of adult equivalents from the fraction of the stock’s total range contained in the WCEEZ multiplied by total adult equivalents in the population, extrapolated from nesting females:

$$\hat{N\prime }=\frac{area\;of\;WCEEZ\;in\;range}{area\;of\;range}\;{\hat{n}}_{2014}\;\hat{RI}\frac{1}{\hat{PF}}{\hat{w}}_{m}{\hat{v}}_{m}$$ (4)

where $\hat{RI}$ is remigration interval (periodicity of nesting in terms of years), $\hat{PF}$ is proportion of females in the population (both in Table 2), and ${\hat{w}}_{m}$ and ${\hat{v}}_{m}$ are stable age distribution and reproductive value vectors (standardized to the values for the adult class) for an estimated Leslie-Lefkovitch transition matrix **A**_*m*_ corresponding to ${\hat{\lambda }}_{m}$ [35]. $\hat{PF}$ was based on the local sex ratio observed in nearshore boat work off California [22], which was similar to the point estimate of 0.75 used in the IUCN Red List assessment for western Pacific leatherbacks [9]. Uncertainty in $\hat{RI}$ was not available, but is likely negligible compared to other sources of uncertainty [56].

**Table 2:** Life history parameters.

Parameter	Symbol (if applicable)	Derivation (if applicable)	Estimated value	Sources
Maximum population growth rate	${\hat{\lambda }}_{m}$		*U*(1.04, 1.06)	See text, S2 Text
Age at first reproduction	*α*		13^1^	[48,52,64]
Adult survival rate	*P*_adult_		*U*(0.88, 0.96)	[52,65–67]
Final sub-adult age class survival rate	*P*_*α*_	Assume equal to *P*_adult_	*U*(0.88, 0.96)	
Erosion (proportion of nests destroyed by beach erosion)			Beta(9.7, 20.2)^2^	[68,29,69]
Emergence success (proportion of eggs in intact nest producing emerged hatchlings)			Beta(2.8, 4.2) ^2^	[68–71]
First-day hatchling survival			0.55	[66]
Survival through first day post-hatching	*P*_hatch_	emergence × (1- erosion) × first-day survival	0.15	See components
Remigration interval	$\hat{RI}$		2.5	[9]
Proportion of females	$\hat{PF}$		Beta(27.5, 10.5) ^3^	[22]
Clutch frequency (nests yr^-1^)	$\hat{CF}$		5.5	[24]
Clutch size (eggs nest^-1^)	$\hat{CS}$		78	[70]
Neophyte^4^ factor (fecundity relative to returning nesters)	*f*_neo_		0.68	[72,73]
Adult fertility	*F*_adult_	$P_{\text{adult}}\times \hat{PF}\times (1/\hat{RI})\times \hat{CF}\times \hat{CS}$	See components	
Neophyte fertility	*F*_*α*_	$P_{\alpha }\times \hat{PF}\times (1/\hat{RI})\times \hat{CF}\times \hat{CS}\times f_{\text{neo}}$	See components	

Symbols, values, and sources for life history parameters estimated from literature review.

1 A fixed *α* was computationally more efficient to model than a distribution. Effect of bias in *α* was evaluated in a sensitivity trial (see Methods).

2 Erosion and emergence success were parameterized such that mean and variance equalled mean and standard error of reported values for JMW.

3 Based on 27 females of 37 turtles off California for whom sex was determined.

4 A neophyte is a first-time nester.

The fraction of the stock’s range in the WCEEZ was calculated as the area of overlap between the WCEEZ [13] and the range of boreal summer nesters, divided by the latter. We approximated the range of boreal summer nesters as the range of the western Pacific leatherback RMU north of 8°S latitude [8,11,12], the approximate southernmost latitude at which turtles from the WCEEZ were observed to return to nest [22] (Fig 1). We used R 3.1.0 and the rgdal [15], sp [16], geosphere [57], and rgeos [17] packages for data import and manipulation and area calculation. The resulting fraction is 1.0%.

We structured **A**_*m*_ as a post-breeding-census [58], Leslie-Lefkovitch transition matrix, which is age-classified but has a single value for survival of adults in the last element of the diagonal [59]. The post-breeding-census approach allows inclusion of eggs and hatchlings in the total reproductive value of the population and thereby allows management of removals of those stages. The resulting matrix has dimensions of age at first reproduction (*α*) plus one. Fertility estimates were taken from published estimates for JMW, but we borrowed information from recovering leatherback populations, primarily in the North Atlantic, to estimate other parameters (Table 2). We fixed ${\hat{\lambda }}_{m}$, adult survival (*P*_adult_), and fertilities for the last juvenile age class and the adult stage (*F*_*α*_ and *F*_adult_) at the means of their estimated probability distributions, approximated survival for the final, sub-adult age class (*P*_*α*_) as equal to the adult survival rate, and solved for the geometric mean survival rate of the remaining juvenile and sub-adult age classes using the characteristic equation (S2 Text). Survival rate is likely an increasing function of size for juveniles [60–62], and size increases asymptotically with age [63], so we modeled age-specific juvenile survival rates with a logarithmic curve (S2 Text). We used sensitivity trials (described below in Step 5) to explore how plausible levels of bias in parameters such as age at first reproduction or survival rates affect population outcomes under the same management regime. Specific parameter estimates and sources are provided in Table 2 [64–73].

#### “Survey” approach

We estimated local abundance from a time series of nearshore aerial surveys off California during leatherback foraging season [46], prorated by the estimated fraction of the year that individuals spend in the WCEEZ annually:

$$\hat{N}=\frac{days\;in\;WCEEZ}{365}\;{\hat{n}}_{\text{CCE},2014}\;{\hat{\varepsilon }}_{LT}$$ (5)

where ${\hat{n}}_{\text{CCE},2014}$ is a forecasted estimate for the time series and ${\hat{\varepsilon }}_{LT}∼N(1,0.14)$ represents systematic error introduced by estimated parameters in the line transect formulae used to estimate abundance from the surveys, which was not accounted for in the probability distribution for ${\hat{n}}_{\text{CCE},2014}$ [46]. The result is only a partial abundance estimate due to the limited spatial extent of the aerial surveys off California relative to the WCEEZ [46] and the existence of additional known foraging grounds off Oregon and Washington [22].

The aerial survey time series is noisy, in part due to oceanographic variability that affects leatherback foraging habitat and behaviour [46], and the last available survey estimate was for 2003. The forecasted estimate ${\hat{n}}_{\text{CCE},2014}$ was obtained by relating the aerial survey time series to the less noisy and more recently updated time series of nest counts at JMW [24] (S1 Text) using a state-space model (S2 Text). This approach accounted for interannual variability in surveyed abundance due to uncertainty introduced by surveys and by environmental influence on spatial distribution, but is predicated on an assumption that the mean proportion of total leatherbacks off California to females nesting at JMW is constant through time.

Based on satellite tag data for seven complete entry-to-exit tracks, days in WCEEZ was estimated as *N*(100,13), i.e., individuals averaged 100 days (SD = 13 days) in the WCEEZ on each visit [data from Scott Benson, courtesy of TOPP and NOAA/NMFS/SWFSC/ERD][74]. Males and females appear to have similar residence times in the WCEEZ [22]. The telemetry data came from satellite tags attached by shoulder harness, which substantially decrease swimming speed [75] and thus may have led to underestimation of days in WCEEZ due to delayed arrival. Satellite tag studies on this population now employ direct attachment.

#### “Tag” approach

We estimated adult female abundance in the WCEEZ based on satellite tag data and remigration interval, then estimated total turtles of all age classes and sexes in the jurisdiction based on local sex ratio and size distributions in CDGN bycatch and at nesting beaches. Local abundance of adult females was estimated as

$${\hat{N}}_{fem,USWCEEZ}\;=\;\frac{days\;in\;WCEEZ}{365}\times proportion\;using\;WCEEZ\times \left(\hat{RI}-1\right)\;{\hat{n}}_{2014}$$ (6)

The proportion using the WCEEZ was estimated as the product of sequential binomial outcomes of nesting females tagged at JMW in boreal summer migrating back to their foraging grounds. This was calculated as Beta(23.5, 14.5) × Beta(10.5, 6.5) × Beta(5.5, 1), (corresponding to 23 of 37 tagged females migrating to the North Pacific [22] × 10 of 16 with sufficiently long tracks to determine region migrating to the northeast Pacific [22] × 5 of 5 with sufficiently long tracks to determine destination within the northeast Pacific migrating to the WCEEZ [data from Scott Benson, courtesy of TOPP and NOAA/NMFS/SWFSC/ERD]). The factor $\hat{RI}-1$ represents the fraction of total adult females in the population not nesting in a given year, and thus expected to make their annual foraging migration to the waters off the west coast of North America. Recent evidence suggests that the remigration interval for leatherbacks foraging in the Northeast Pacific is longer than for other foraging groups, so using the average remigration interval for the nesting population will likely bias the local abundance estimate downward.

Total leatherback abundance in the WCEEZ was then estimated as

$$\hat{N}\;=\frac{1}{proportion\;adults}\;\frac{1}{\hat{PF}}\;{\hat{N}}_{fem,USWCEEZ}$$ (7)

where the adult fraction was estimated from observer data for the CDGN fishery, calculated as the proportion of measured turtles longer than the mean curved carapace length of boreal-summer-nesting females at JMW minus one standard deviation (i.e., 148.8 cm) [22]. Two of four measured turtles exceeded this length, so the proportion was estimated as a one-sided truncated probability distribution, Beta(2.5, 2.5) [48], with a lower bound of 0.23 to account for age classes unlikely to occur in the WCEEZ [47]. If males remigrate more frequently than females, the local abundance estimate would be biased only slightly upward, since males constitute a small proportion of the population.

### Step 5: Tune LRP estimator for uncertainty and risk tolerance

The goal of this step is to identify values for the tuning parameters of the LRP estimator at which population conservation objectives are met within the specified risk tolerances, in the face of realistic uncertainties. For each LRP estimation approach, we conducted MSE base trial simulations to characterize the performance of the LRP estimator as a removal limit over a range of percentiles of the estimated LRP probability distribution and values for *f*_a_. We used the results to tune the estimator (i.e., find a combination of *f*_a_ and percentile levels) to satisfy the specified performance criteria (i.e., maintain or rebuild populations above *N*_MNP_ with 95% success rate, and above *N*_collapse_ with 97.5% success rate). Base trial simulations were also used to characterize performance of the LRP estimator with respect to the expedited rebuilding objective. Sensitivity trials were used to evaluate sensitivity of performance to plausible biases in key model assumptions and parameters.

The MSE simulations consisted of three parts. A biological (“true”) operating model simulated leatherback population dynamics. An observation (“estimation”) model simulated imperfect measurement and estimation of biological variables and parameters (e.g., abundance and reproductive value). The estimates were used in a management model that simulated estimation and application of the LRP as an output control rule (i.e., limiting annual mortality to the LRP). Resulting human-caused mortality then fed back into the operating model. For each combination of percentile of the LRP distribution and value of *f*_a_ evaluated, risk of management failure (i.e., not satisfying performance criteria) was estimated through Monte Carlo simulations (*n* = 2000) that reflected uncertainties in our knowledge of life history parameters, in abundance and catch estimation, and due to environmental stochasticity and other important factors [5].

Performance evaluation of a local LRP as a control rule requires assuming that the entire population throughout its range is similarly managed. In this case, given the assumed lack of selectivity of the CDGN with respect to available sizes in the WCEEZ, this means that in the MSE, all age classes for the entire population incur the same (LRP-limited) human-caused mortality.

The age-structured framework for this approach is described in detail in Curtis and Moore [35]. The biological, observation, and management models used here are outlined in S3 Text, noting where we have made modifications and how application varied among the “naïve”, “survey”, and “tag” approaches to LRP estimation. All simulations and analyses were conducted in R 3.1.0 [14] with RStudio [76], using the popbio [77], MASS [78], gtools [79], and abind [80] packages. Code for R functions used for the MSE is provided in S5 Text.

#### Base trial details

For each of the three LRP estimation approaches, three sets of base trials were run to assess the risk of failing to satisfy each of the three conservation objectives over a range of LRP percentiles and values for *f*_a_. A stable age distribution would be the long-term expectation for a population subjected to constant bycatch mortality with age, so every simulated population was initialized at its stable age distribution according to the corresponding random realization of **A**_*m*_.

For the *N*_MNP_ and *N*_collapse_ objectives, management outcomes were evaluated at the end of 40 years (approximately two generations), for simulations with LRP percentiles ranging from the 2.5^th^ to the 50^th^, and with two values for *f*_a_: 1 and 0.6. Setting *f*_a_ = 1 is a default [6,35], and *f*_a_ = 0.6 was chosen because preliminary results indicated that when *f*_a_ = 1, the performance criterion for the *N*_MNP_ threshold was met only at very low percentiles in the tail of the LRP probability distribution, where estimates are relatively unstable. Using *f*_a_ = 0.6 allowed for more stable LRP estimates. We identified the highest percentile and *f*_a_ value at which populations met the performance criteria. Risk was measured as percentage of populations (i.e., simulations) with a final abundance of adults less than their initial abundance.

The third set of base trial simulations evaluated LRP performance over 200 years in terms of relative time required for population rebuilding from 0.1*K* to *N*_MNP_. LRPs were evaluated at ten *f*_a_ values from 0.1 to 1, using the LRP percentile identified from the base trials for the *N*_MNP_ and *N*_collapse_ objectives. Risk was measured as percentage of populations with rebuilding times more than 10% longer than the time required for a population free of human-caused mortality. Populations were considered rebuilt if abundance of adults was at least that of a population with a stable age distribution at *N*_MNP_.

#### Sensitivity trials

Sensitivity trials explored effects on LRP estimator performance of important plausible biases in the observation and management models relative to the biological model. Performance was evaluated with respect to the *N*_MNP_ and *N*_collapse_ thresholds.

Four sensitivity trials were run for the *N*_MNP_ threshold to explore the effect of bias in the observation model: (1) age at first reproduction was underestimated, with true *α* = 20 [9,81]; (2) natural juvenile survival rates increased more steeply in the first few years than estimated, but had the same geometric mean; (3) fertility was underestimated by a factor of two at the same ${\hat{\lambda }}_{\text{m}}$; and (4) adult survival was underestimated at the same ${\hat{\lambda }}_{\text{m}}$, with true *P*_adult_ ~ *U*(0.93,0.97). Details for implementation of the first two sensitivity trials are provided in S4 Text.

For the *N*_collapse_ threshold (or maintaining current numbers of adults), an additional sensitivity trial was run using an unstable starting age distribution with more adults relative to the rest of the population than in the stable age distribution (details in S4 Text). Finally, applicable to the objective of avoiding a decrease in adult abundance (but not relevant to the *N*_collapse_ threshold), a sensitivity trial was run using a starting point for the populations of 0.15*K* (instead of 0.1*K*), an upper estimate based on more reliable–but later (i.e., likely after some population decline took place)–nest counts from JMW [24].

## Results and Discussion

### Abundance and productivity estimates

The projected estimate of mean females nesting annually, ${\hat{n}}_{2014}$, was 318 (90% CI of 302 to 334). Corresponding estimates of local abundance from the three approaches are provided in Table 3. Differences in uncertainty among these abundance estimates drive the differences in uncertainty among the corresponding LRP. As noted earlier, the “tag” approach is the most appropriate for inference in the current study.

**Table 3:** Local abundance estimates.

Approach	Median abundance (95% CI)	CV	Units
Naïve	32.6 (27.4, 41.5)	11%	adult equivalents
Survey	15.4 (8.0, 27.9)	33%	individuals
Tag	109 (40.2, 278.9)	50%	individuals

Estimates of annual abundance of leatherback turtles in WCEEZ in 2014 for three approaches used to calculate local LRPs (pro-rated for the fraction of the year in which they occur there for the “survey” and “tag” approaches).

Although the “naïve” estimate of adult equivalents in the WCEEZ has the lowest CV of the three, it does not incorporate uncertainty in relative abundances and reproductive values of different age classes (which affect the estimate of adult equivalents), nor does it incorporate uncertainty in the range of boreal summer nesters. Were this the best available approach, further work on accounting for these sources of uncertainty would be important.

Uncertainty in the “survey” estimate is dominated by the conversion factor from nesting beach counts at JMW to nearshore survey estimates off California, which reflects not only survey uncertainty but also high interannual variability in habitat use [46]. The “survey” estimate is much lower than the “tag” estimate, which is reasonable given the limited spatial extent of the aerial surveys off California relative to the WCEEZ [46] and the existence of additional known foraging grounds off Oregon and Washington [22].

The two main sources of uncertainty in the “tag” abundance estimate are the proportion of females nesting at JMW that migrate to foraging grounds in the WCEEZ, and the proportion of leatherbacks within the WCEEZ that are adults. Increasing the number of satellite tags attached to females at nesting beaches would reduce uncertainty. Stable isotope techniques, which are cheaper to collect than satellite tag data, may also help improve predictions of large-scale movements such as foraging ground destination, especially as their specificity, accuracy, and power improve, as exemplified by compound-specific isotope analysis [30,54]. The greatest reduction in uncertainty for the “tag” approach would be achieved by improving information on the adult fraction of turtles in the WCEEZ. Nearshore boat work [22] and strandings may provide biased size samples, for example if younger turtles remain in warmer offshore waters. Requiring fisheries observers in the CDGN fleet to measure entangled leatherbacks would provide valuable information, albeit slowly at the current low interaction rate [10].

The estimate for ${\hat{\lambda }}_{m}$ was based on recovering leatherback populations nesting in the northwestern Atlantic and South Africa. Although reproductive output for the North Atlantic and western Pacific populations is similar, young juveniles foraging in warmer, less productive Pacific waters could have lower survival and growth rates than their counterparts in the Atlantic [55], leading to lower population productivity. This potential bias may be offset by the fact that productivity estimates for the Atlantic and South African nesting beaches were likely somewhat supressed by human-caused mortality. In any case, direct estimates of survival rates, human-caused removals, and age at first reproduction for the western Pacific population would improve the estimate of potential productivity.

Possible metapopulation dynamics of western Pacific leatherbacks are an unaccounted-for source of uncertainty in abundance and productivity estimates. We assumed that turtles from different boreal-summer-nesting foraging destinations contribute equally to each other’s abundance over the long term through mixing during breeding and nesting and chance dispersal by ocean currents, effectively integrating productivity across foraging groups that vary in foraging habitat quality and migration distance [54]. If the population is more structured, the time series at JMW may not be representative of relative abundance with time in the WCEEZ. Updated aerial survey estimates off California would be informative in this regard (S2 Text).

We also assumed zero exchange between populations of boreal summer and winter nesters. Given the continuity in nesting between peak seasons [29], this assumption is likely to be violated. However, since the “survey” and “tag” approaches directly predict abundance within the WCEEZ proportional to abundance at JMW, and the summer and winter time series at these beaches have similar trends [24], some mixing would be unlikely to substantially affect the resulting abundance estimates.

### Management Strategy Evaluation

#### Base trial results

**Fig 2:**
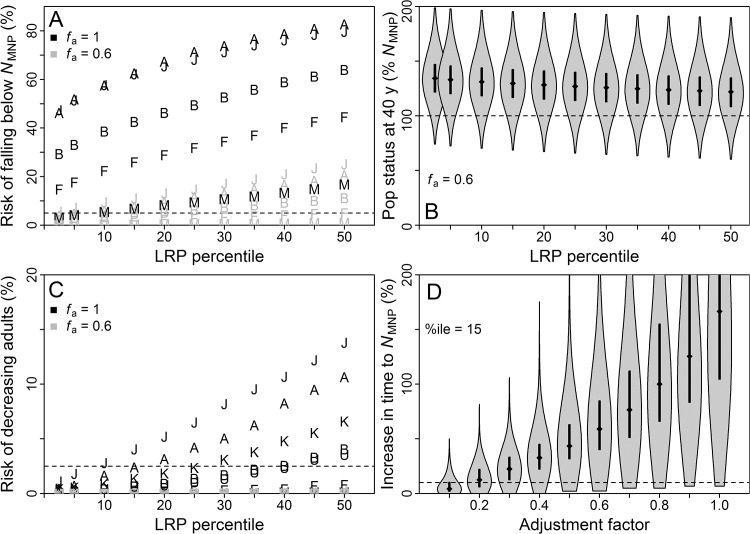
Results of simulations for “naïve” approach. Results from base and trial simulations, for population growth under management based on “naïve” approach to local limit reference point (LRP) estimation. Population outcomes are presented in terms of abundance of adults relative to that at *N*_MNP_ for a stable age distribution. (A) Population outcomes in terms of probability of being below *N*_MNP_ after 40 years, after starting at *N*_MNP_ and using a range of LRP percentiles. Dashed line indicates 5% risk. Symbols correspond to trial: B = base, A = underestimated adult survival, J = underestimated initial steepness of juvenile survival-with-age, F = underestimated fertility, M = underestimated age at first reproduction. (B) Violin plot (combined boxplot and kernel density plot) of probability distribution for population outcomes after 40 years, after starting at *N*_MNP_ and using a range of LRP percentiles. Medians and first and third quartiles shown in black for each case. (C) Population outcomes in terms of probability of adults decreasing from initial abundance after 40 years, after starting at 0.1*K* and using a range of LRP percentiles. Dashed line indicates 2.5% risk. Symbols correspond to trial, as above, plus K = underestimated current proportion of *K* (this trial was started at 0.15*K*), U = unstable starting age distribution. (D) Violin plot of probability distribution for rebuilding times from 0.1*K* to *N*_MNP_, relative to rebuilding times for the same populations without bycatch mortality, at the highest common LRP percentile meeting both performance criteria (based on panels A and C). Dashed line indicates 10% increase in rebuilding time. Medians and first and third quartiles shown in black for each case. Figure created in R 3.1.0 [14] with the help of the vioplot [82] and extrafont [20] packages.

**Fig 3:**
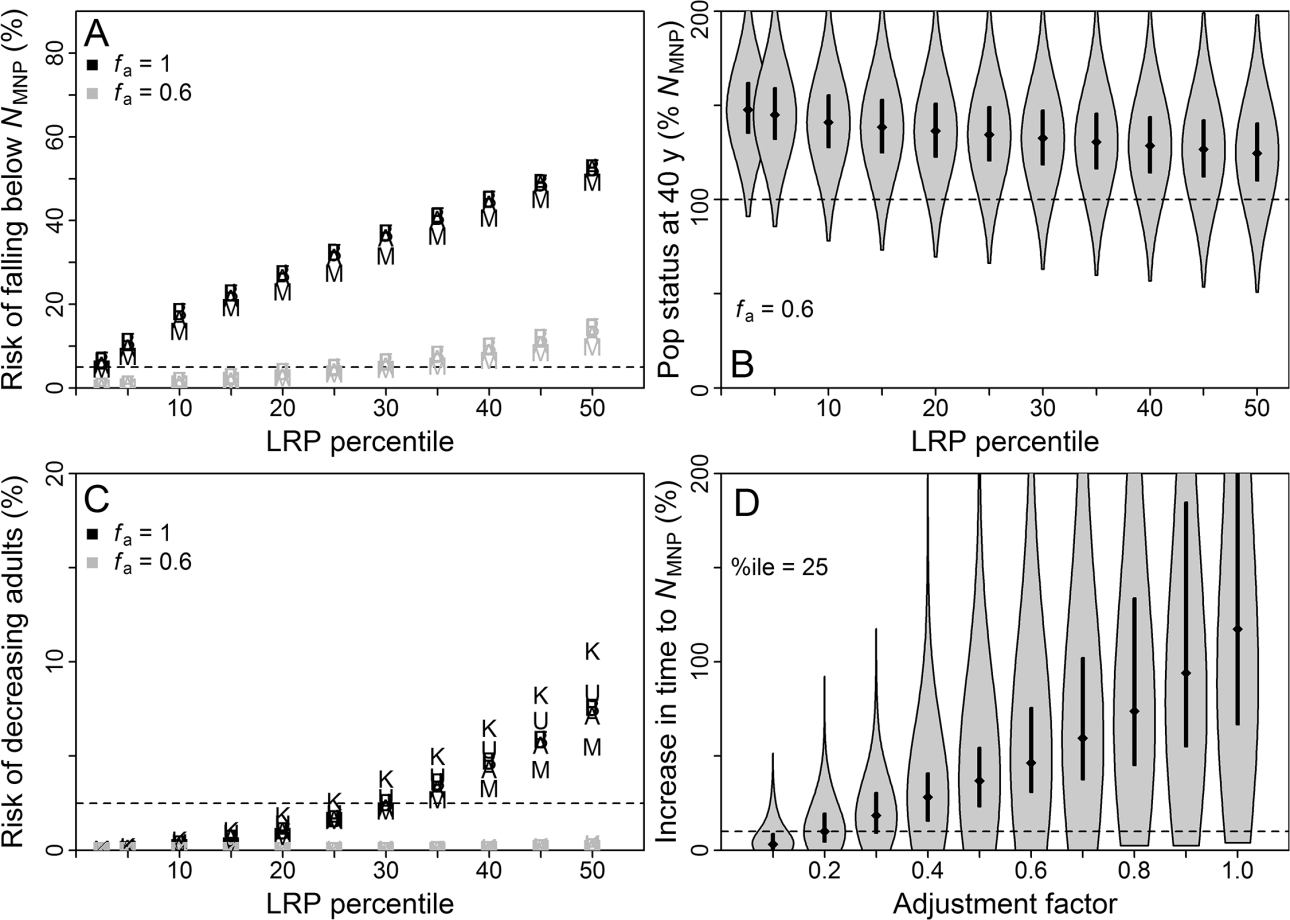
Results of simulations for “survey” approach. Results from base and trial simulations, for population growth under management based on “survey” approach to local LRP estimation. Population outcomes are presented in terms of abundance of adults relative to that at *N*_MNP_ for a stable age distribution. All panels as described for Fig 2. Figure created in R 3.1.0 [14] with the help of the vioplot [82] and extrafont [20] packages.

**Fig 4:**
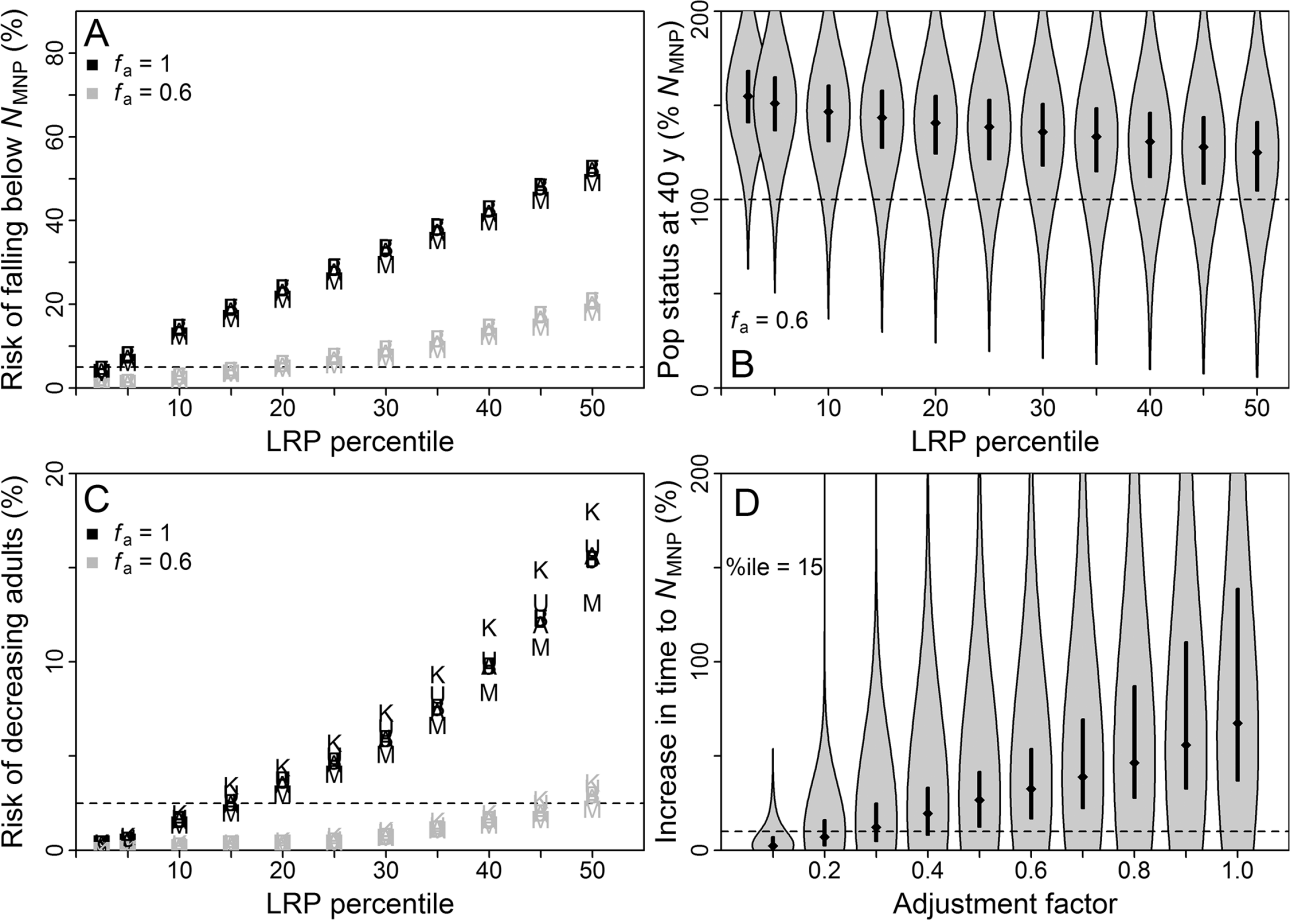
Results of simulations for “tag” approach. Results from base and trial simulations, for population growth under management based on “tag” approach to local LRP estimation. Population outcomes are presented in terms of abundance of adults relative to that at *N*_MNP_ for a stable age distribution. All panels as described for Fig 2. Figure created in R 3.1.0 [14] with the help of the vioplot [82] and extrafont [20] packages.

An *f*_*a*_ of 1 was insufficient to meet the performance criterion for the primary (*N*_MNP_) objective for all three approaches to local abundance estimation. For *f*_a_ = 0.6, the performance criterion was met when using the 15^th^ percentile of the LRP distribution for the “naïve” abundance approach, the 25^th^ percentile for “survey”, and the 15^th^ percentile for “tag” (Figs 2–4A). For all three approaches and across all LRP percentiles considered, more than 75% of simulated populations exceeded *N*_MNP_ at *f*_a_ = 0.6 (Figs 2–4B). The performance criterion for the safeguard (*N*_collapse_) objective was met across nearly all LRP percentiles for all three approaches (Figs 2–4C).

Population outcomes for the “naïve” approach were considerably worse than for the other approaches for *f*_a_ = 1 (Figs 2–4A), reflecting uncertainty in relative abundance and reproductive value of age classes that was not accounted for in the LRP equation. Future applications of the RVLL tool for LRP estimation may benefit from including uncertainty in the underlying transition matrix in the LRP equation.

The third conservation objective evaluated was to expedite rebuilding to *N*_MNP_. For the “survey” and “tag” approaches, setting *f*_a_ = 0.1 and using the LRP percentile dictated by the *N*_MNP_ maintenance criterion resulted in more than 75% of populations rebuilding within 10% of the time required by an unimpacted population (Figs 2–4D). For the “naïve” approach, close to 75% of populations achieved this objective. A 95% probability of staying within that timeframe, which was used to tune PBR application to threatened and endangered populations of marine mammals [6], would require *f*_a_ < 0.1.

#### Sensitivity trial results

Performance of LRPs estimated using the “naïve” approach proved more sensitive than the other approaches to biases in information (Figs 2–4A and 4C) due to estimation bias in reproductive value that affects RVLL but not PBR estimates. For example, populations managed under the “naïve” approach fared better when age at first reproduction was underestimated with ${\hat{\lambda }}_{m}$ fixed, because this led to underestimation of true total abundance and reproductive value in the population.

Performance of LRPs estimated using the “survey” and “tag” approaches was not sensitive to most biases explored. Exceptions were underestimation of current percentage of carrying capacity and an unstable initial age distribution skewed towards adults, which both increased risk of adults declining from their current level, likely due to demographic effects rather than bias in LRP estimation. Underestimating age at first reproduction led to somewhat improved population outcomes relative to base trials (Figs 3 and 4C). Population outcomes for the “survey” and “tag” approaches were more robust to bias because the local abundance estimates were based on empirically measured age distributions in the study area rather than on assumptions about population age structure, as in the “naïve” approach.

### LRP estimates and considerations for real-world application

**Fig 5:**
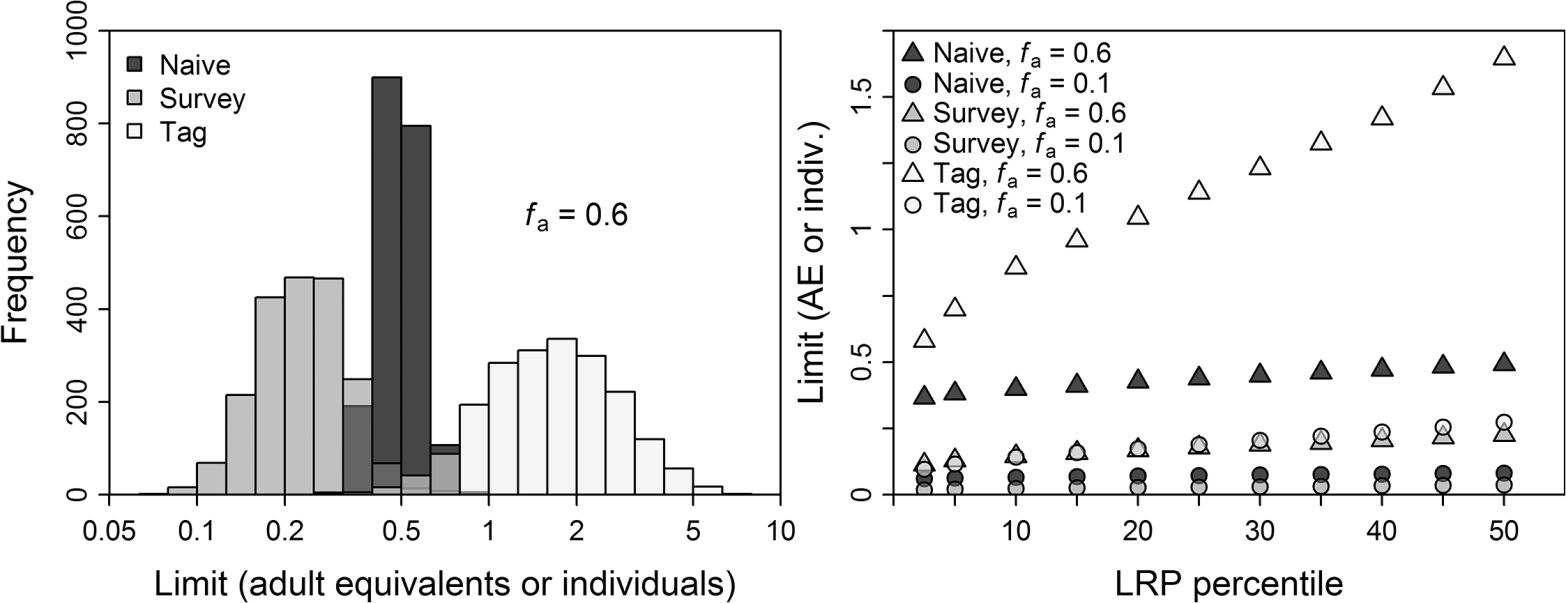
LRP estimates for three approaches. Calculated local LRP distributions (left) and percentiles (right) for 2014 using each of three approaches to local LRP estimation: “naïve” (LRP in terms of adult equivalents), “survey” (LRP in terms of individuals), and “tag” (LRP in terms of individuals). Figure created in R 3.1.0 [14] with the help of the extrafont [20] package.

The LRP estimate for human-caused removals in the WCEEZ based on the “tag” approach and the *N*_MNP_ objective was 4.7 leatherbacks per five years (Fig 5, right panel). If *f*_a_ is set to 0.1 to expedite population rebuilding, that limit drops to 0.8 leatherbacks per five years. These removal LRPs convert to 6.9 and 1.2 interactions per five years, using an expected discard mortality of 0.68 deaths per turtle taken in the CDGN [38]. If solely the *N*_collapse_ objective were applied, an LRP of 7.7 removals (11.3 interactions) per five years would satisfy the performance criterion (Figs 4C and 5B). By comparison, the most recent (2013) Biological Opinion for the CDGN concluded that a removal level of seven leatherbacks per five years (i.e., 10 interactions per five years) was “not likely to appreciably reduce the probability of survival or recovery of the species” [38]. In actuality, the population has declined substantially over the period included in the Incidental Take Statement, and a recent estimate of current leatherback removals by the CDGN has a probability distribution centered well below five turtles per five years [10]. Comparing the LRP estimates to the jeopardy assessment is difficult, however, since the analyses are not based on the same criteria. If the LRP approach demonstrated in this analysis were incorporated into management of leatherback bycatch within the context of the ESA, conservation objectives and risk tolerances–and thus the resulting LRP values–would need to be revisited and perhaps revised.

We have presented 5-year rather than annual LRP estimates, because a multi-year evaluation period is appropriate where LRPs are low or taxa have “slow” life histories in order to smooth stochastic effects (e.g., due to process and observation error) on the management process. Annual estimates of rare-event bycatch based on low observer coverage are prone to high levels of sampling error [83]. This, along with stochastic variation in true catches, can lead to volatile and costly management if assessments and management decisions are conducted annually, without providing additional conservation benefit. Basing decisions on evaluation of longer-term mean catch estimates will increase fishery and management stability, thereby reducing management and fishery costs.

The LRP estimates come with important caveats related to uncertainty in human-caused removals, in addition to those related to abundance estimation (see “Abundance and productivity estimates” above), which should be considered if LRPs are incorporated into management.

**Fig 6:**
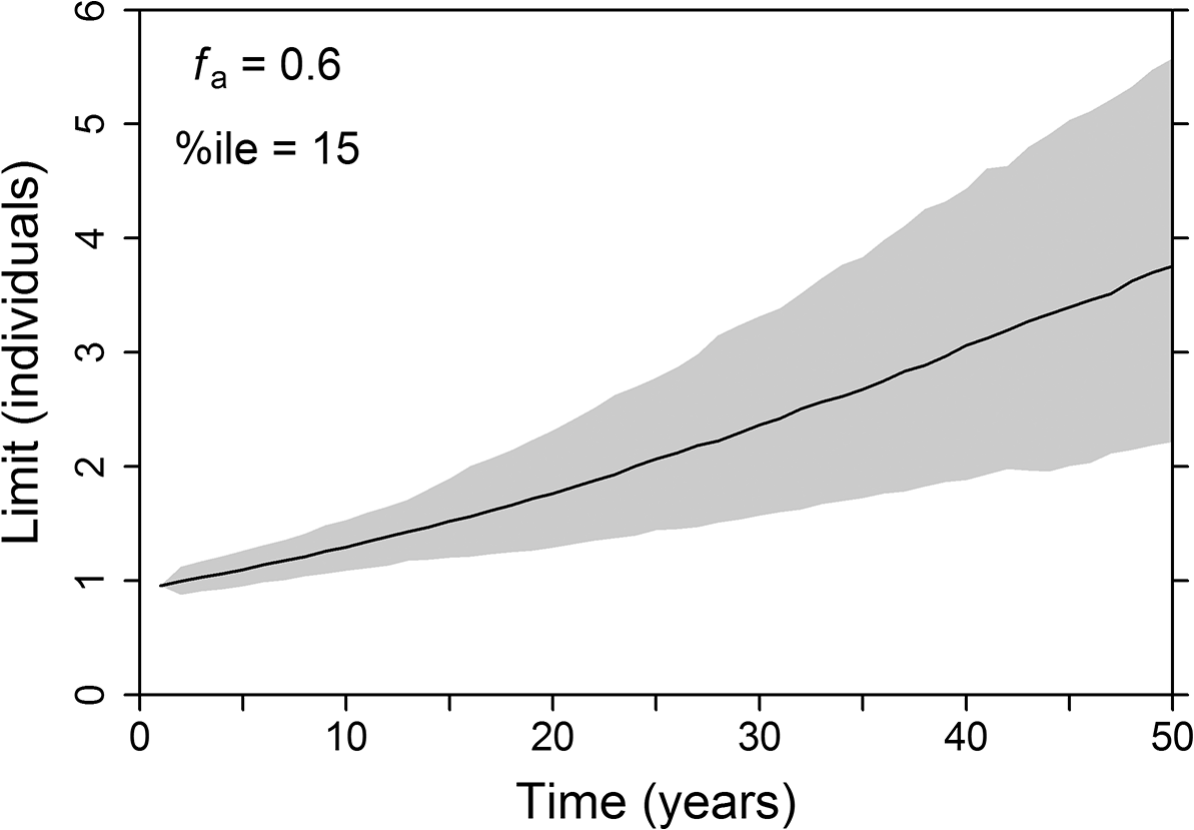
Projected local LRP with population-wide management. Projected local LRPs (median and 90% confidence intervals) for a population if the entire population were managed based on LRPs, calculated with the “tag” approach. Figure created in R 3.1.0 [14] with the help of the extrafont [20] package.

- **Other sources of human-caused mortality.** The MSE was based on bycatch in the CDGN being the sole source of human-caused mortality in leatherbacks in the WCEEZ. In reality, other human activities also lead to leatherback removals within the jurisdiction, including vessel strikes and interactions with other fisheries. From 2008 to 2012, the National Marine Fisheries Service West Coast Region California Sea Turtle Stranding Database lists two leatherbacks determined to be killed by direct human-related causes other than the CDGN, and another six whose cause of death was illness or undetermined [84] (R. LeRoux, NOAA Southwest Fisheries Science Center, USA, pers. comm.). LRPs pertain to cumulative human-caused mortality from all sources, not just fisheries, but most sources are not quantified. Moreover, documented strandings likely substantially underestimate true removals of leatherbacks [85], so better estimates of carcass recovery and determinations of cause of death are needed to assess total human-caused removals.
- **Management uncertainty.** We assumed in the MSE that annual human-caused mortality was known perfectly (S3 Text). This was a practical decision due to the challenges of modeling realistic observation error when bycatch is a rare event, for example, it would vary with observed bycatch and thus population size [10]. In reality, annual coverage in the CDGN averages 15.6% [10], while other sources of human-caused mortality, such as vessel strikes, are not quantified at all. We therefore recommend a risk-based approach that estimates the probability of exceeding the LRP over multi-year (e.g., 5- to 10-year) evaluation periods.
- **Fishery selectivity.** Size-selectivity of the CDGN (or other sources of human-caused mortality) due to spatial segregation of size classes may also introduce bias in the PBR-based “survey” and “tag” approaches, which assume that all stages in the management area are removed at equal rates. Likewise, sex-selectivity might result in a greater or lesser effect on the population at the same mortality [86].
- **Local LRP, global evaluation.** While we necessarily assume in the MSE that human-caused mortality is the same across regions and age classes, since it is evaluating a management strategy for a non-selective fishery on part of the population, this is of course not true in reality. The MSE-tested local LRP provides a valid measure of a jurisdiction’s local management responsibility within an international cooperative management scheme. This cooperation is required for the population to achieve the specified conservation objective(s), with population increases accompanied by increases in local LRPs (Fig 6). If local LRP control rules are applied unilaterally and the population’s status is driven by impacts elsewhere, then maintaining removals below the LRP may not help rebuild the population, nor will removals exceeding the LRP necessarily have much of an impact on extinction risk. Rather, local LRPs in this case are useful as benchmarks for indicating whether local impact is out of proportion with local abundance.

## Conclusions: benefits of LRP-based management

Moving towards LRP-based management of leatherbacks and other marine megafauna may have significant conservation benefits. LRPs provide a clear and rational basis for evaluating current management of human impacts on species of conservation concern. While control rules based on local LRPs may seem to yield trivial short-term conservation benefits in some cases, a local LRP scheme provides a basis for quantifying local responsibility for shared resources, advancing population-based management in other jurisdictions to achieve incremental progress, and coordinating population management across jurisdictions. LRPs, including local LRPs for highly mobile species such as leatherbacks, also provide a standard metric that could serve as a basis for certification, for example to gauge whether international fisheries meet U.S. management standards for seafood imports under the Magnuson-Stevens Fishery Conservation and Management Act, or for the Marine Stewardship Council.

Our local LRP estimates suggest that if the current human-caused removal rate for leatherbacks in the WCEEZ were applied to the entire boreal-summer-nesting western Pacific population, the population could likely rebuild to *N*_MNP_ eventually. However, the certainty of this conclusion is compromised by not knowing how much mortality occurs in the WCEEZ from sources other than the CDGN. In any case, rebuilding would occur at a slower rate than set forth by the expedited-rebuilding objective. In reality, the western Pacific leatherback population will continue to decline until impacts to turtles in jurisdictions outside the U.S. are reduced. Local LRPs could be used to identify where these impacts are the greatest and determine how much mitigation is needed, and thereby guide priorities for international conservation action.

## Supporting Information

S1 Text Assessment of population status. (PDF)

S2 Text Supporting information on parameters for life history and abundance estimation. (PDF)

S3 Text Supporting information on MSE: biological, observation, and management models. (PDF)

S4 Text Supporting information on sensitivity trial methods. (PDF)

S5 Text R functions for MSE. (PDF)
