# Supplementary material for: Estimating Limit Reference Points for Western Pacific Leatherback Turtles (Dermochelys coriacea) in the U.S. West Coast EEZ
Source: PLoS One. 2015 Sep 14;10(9):e0136452. doi: 10.1371/journal.pone.0136452 (PMC4569282; doi:10.1371/journal.pone.0136452)
Supplement: S1 Text — (PDF) [file pone.0136452.s001.pdf]

S1 Text. Assessment of population status

We categorized population status for boreal-summer-nesting western Pacific leatherbacks in terms of IUCN Red List Criterion E, which requires a quantitative assessment of extinction risk. Extinction risk was approximated as the probability of crossing a quasi-extinction threshold, defined here as 20 mature individuals, in the next 100 years [1]. To evaluate extinction risk, we fit a linear state-space model to time series of boreal-summer-nesting females. We used bootstrapped values of the resulting estimates of population growth rate, observation error, and process error to project nest counts 100 years into the future, and converted those counts to total adult abundance to assess extinction risk.

Population time series

Time series of nest counts from Jamursba Medi and Wermon (JMW) beaches in West Papua, Indonesia provide the best available information on abundance of boreal summer nesters over time [2] and represent the vast majority of nesting for boreal summer nesters. The time series for boreal summer at Jamursba Medi runs from 1993 to 2011, with one missing year (1998), and consists of "adjusted" nest counts as reported in Tapilatu et al. [2]. We used a corrected value for 2011 of 1516 nests (R. Tapilatu, State University of Papua, Indonesia, pers.comm.). Nest counts for Jamursba Medi were converted to nesting females by dividing by 5.5, the mean nests per female observed at JMW [2]. To obtain a time series for boreal summer nesters at Wermon, the number of nesting females in boreal summer at Jamursba Medi was subtracted from estimates of total females nesting in boreal summer , available from 2005 to 2011 [2], so values for 1993 to 2004 were missing in the Wermon time series.

State-space model

The two time series were log-transformed and fit with a Multivariate Auto-Regressive State-Space (MARSS) model using the MARSS package in R [3,4]. The underlying population process was modeled as a single, common, hidden state, represented by

$$x_t = x_{t-1} + u + w_t \text{ , where } w_t \sim \text{N}(0, q) \quad \text{(S1.1)}$$

where $\boldsymbol{x}$ is a 1 × 19 matrix of population states, u is population growth rate (a scalar), and w_t is process error in year t with process variance q. The two time series are observations of the underlying process, and are represented by

$$\mathbf{y}_t = x_t + \mathbf{a} + \mathbf{z}_t \text{ , where } \mathbf{z}_t \sim \text{MVN}(0, \mathbf{R}) \quad \text{(S1.2)}$$

where **y** is a 2 × 19 matrix of log-transformed observations, **a** is a 2 × 1 matrix of relative scaling parameters, and **z** is a 2 × 19 matrix of observation errors with 2 × 2 covariance matrix **R**. The scaling parameter for the first time series, at Jamursba Medi (a_1), is fixed at zero in the model fitting process, so and scaling parameters for the remaining time series (here, a_2, for Wermon) are fit relative to that. The observation errors in this model include variability in the proportion of the population nesting each year, variability in the use of each nesting beach, and error in nest counts. Error in the estimate of mean nests per female was trivial in comparison to interannual variability in nest counts [2].

Four models, varying in how **R** was modeled, were fit to the data: independent observation errors in each time series with equal variances, independent errors with unequal variances, covarying errors with equal variances, and covarying errors with unequal variances. Each model was fit via maximum likelihood using a constrained expectation-maximization algorithm [5], which is robust to missing values [6], with 1000 Monte Carlo draws of initial conditions. We evaluated relative model fits with a version of Akaike's Information Criterion

designed for state-space models, AICb, which estimates the penalty term based on bootstrapping [7,8]. The simplest model, with independent observation errors with equal variances, fit best, with an AICb value of 10.0. The next best fit was the model with independent observation errors with unequal variances, with an AICb value of 15.1. The estimates for u and q for the two models were similar.

Uncertainties for parameter estimates were obtained from 2500 bootstraps of the best-fit model. Estimates (and bootstrapped 95% confidence intervals) were: –0.059 (–0.078, –0.040) for $\hat{u}$, 0.00 (0.00, 0.00) for $\hat{q}$, 0.055 (0.023, 0.080) for the nesting beach observation variance, and -1.36 (-1.59, -1.13) for $\hat{a}_2$. The estimate for $\hat{u}$ was similar to that of Tapilatu et al. [2] for nesting leatherbacks at JMW in both seasons. Although an estimate of zero for process variance is no doubt too low, it is likely that most of the interannual variance in nesting is unrelated to true fluctuations in total number of adults in this relatively long-lived species, instead being dominated by interannual variation in the fraction of females nesting, which falls under observation error. Underestimating $\hat{q}$ leads to underestimating uncertainty in projected population size.

Forecasting extinction risk

The underlying state was projected stepwise from the estimated state at the end of the time series, $\hat{x}_{2011}$, for 103 years to achieve an effective forecast of 100 years as of 2014, by calculating

$$\hat{x}_t = \hat{x}_{t-1}\, e^{\hat{u}+\hat{w}_t}\text{, where } \hat{w}_t \sim \mathrm{N}(0, \hat{q}) \qquad \text{(S1.3)}$$

where $\hat{u}$ is the bootstrapped estimate for population growth rate over the time series, and $\hat{w}_t$ is process error with bootstrapped estimate for variance $\hat{q}$.

The estimate of mean total nesting females for each forecast year was calculated as

$$\hat{n}_t = e^{\hat{x}_t}\left(e^{\hat{a}_1} + e^{\hat{a}_2}\right) \tag{S1.4}$$

where $\hat{a}_1$ and $\hat{a}_2$ are bootstrapped estimates for the scaling parameters. Estimated total abundance of mature individuals in the population was calculated as a product of $\hat{n}_t$ (a distribution) and point estimates of remigration interval (2.5) and the reciprocal of the proportion of females in the population (1/0.75) used in the IUCN assessment for the western Pacific leatherback RMU [9]. Probability of extinction at each time step was the percent of projected population trajectories falling below the quasi-extinction threshold of 20 mature individuals.

The population crossed the 50% threshold of extinction probability at 68 years into the future (year 71 of the projection). Given the 30-year generation time used in the formal IUCN assessment for western Pacific leatherbacks [9], which meets the requirements of Critically Endangered classification under IUCN Red List Criterion E. The timing of crossing the 50% threshold is not sensitive to change in process error, so even with a process variance of 0.03 in every simulation, the population would still have qualified as Critically Endangered.

References

1. IUCN Standards and Petitions Subcommittee. Guidelines for using the IUCN Red List categories and criteria, Version 10. Prepared by the IUCN Standards and Petitions Subcommittee. [Internet]. 2013. Available: http://www.iucnredlist.org/documents/RedListGuidelines.pdf

2. Tapilatu RF, Dutton PH, Tiwari M, Wibbels T, Ferdinandus HV, Iwanggin WG, et al. Long-term decline of the western Pacific leatherback, *Dermochelys coriacea*: a globally important sea turtle population. Ecosphere. 2013;4: art25. doi:10.1890/ES12-00348.1

3. Holmes EE, Ward EJ, Wills K. MARSS: Multivariate autoregressive state-space models for analyzing time-series data. R J. 2012;4: 11–19.

4. R Core Team. R: A language and environment for statistical computing [Internet]. Vienna, Austria: R Foundation for Statistical Computing; 2014. Available: http://www.R-project.org/

5. Holmes EE. Derivation of the EM algorithm for constrained and unconstrained marss models. Northwest Fisheries Science Center, Mathematical Biology Program; 2012.

6. Holmes EE, Ward EJ, Scheuerell MD. Analysis of multivariate time-series using the MARSS package [Internet]. NOAA Fishereis, Northwest Fisheries Science Center, 2725 Montlake Blvd E, Seattle, WA 98112; 2014. Available: http://cran.r-project.org/web/packages/MARSS/vignettes/UserGuide.pdf

7. Cavanaugh JE, Shumway RH. A bootstrap variant of AIC for state-space model selection. Stat Sin. 1997;7: 473–496.

8. Ward EJ, Chirakkal H, González-Suárez M, Aurioles-Gamboa D, Holmes EE, Gerber L. Inferring spatial structure from time-series data: using multivariate state-space models to detect metapopulation structure of California sea lions in the Gulf of California, Mexico. J Appl Ecol. 2010;47: 47–56. doi:10.1111/j.1365-2664.2009.01745.x

9. Tiwari M, Wallace BP, Girondot M. *Dermochelys coriacea* (West Pacific Ocean subpopulation) [Internet]. 2013. Available: <www.iucnredlist.org>
