# Supplementary material for: Estimating Limit Reference Points for Western Pacific Leatherback Turtles (Dermochelys coriacea) in the U.S. West Coast EEZ
Source: PLoS One. 2015 Sep 14;10(9):e0136452. doi: 10.1371/journal.pone.0136452 (PMC4569282; doi:10.1371/journal.pone.0136452)
Supplement: S2 Text — (PDF) [file pone.0136452.s002.pdf]

S2 Text. Supporting information on parameters for life history and abundance estimation

Life history parameters

Maximum population growth rate

To adjust the highest mean population growth rate estimated for St. Croix, 13% [1], for augmentation due to the nesting protection program, we populated a post-breeding transition matrix with published life history parameters for St. Croix, including fertility as a product of per capita hatchling production reported for 2001, the reciprocal of remigration interval, proportion of females in the population, and adult survival [1–3]. We estimated the eigenvalue for this transition matrix; then replaced the fertility term with the product of the point estimates of fertility used in the post-breeding census matrix (which equals per capita egg production), probability of a nest avoiding destruction by erosion, and emergence success for boreal summer nesters (Table 2); and estimated the resulting eigenvalue. Predation is an important source of egg mortality, but was omitted from the calculation of maximum productivity because it is largely associated with domesticated animals, and thus not considered natural mortality [4–7]. We subtracted the difference between eigenvalues from 13% to obtain the upper limit of possible growth rates for boreal summer nesters, 6%.

Juvenile survival rate

We solved for the product of survival rates for age classes zero through $\alpha-1$ by simplifying and rearranging the characteristic equation [8]:

$$\prod_{i=1}^{\alpha-1} P_i = \frac{1 - \hat{\lambda}_m^{\;-1} P_{\text{adult}}}{\hat{\lambda}_m^{\;-\alpha} F_\alpha - \hat{\lambda}_m^{\;-(\alpha+1)} P_{\text{adult}} F_\alpha + \hat{\lambda}_m^{\;-(\alpha+1)} P_\alpha F_{\text{adult}}} \tag{S2.1}$$

where P_i is the survival rate of age class i. Egg and first-day, neritic, hatchling mortality are high relative to pelagic mortality and driven by different processes, so we divided the product of juvenile survival rates by the expected survival rate from laying through the first day post-hatching (P_{hatch}; Table 2) before modeling survival with age. We estimated age-specific juvenile mortality as

$$P_i = P_\alpha \left(\frac{i}{\alpha}\right)^b, \text{ where } b = \frac{(\alpha-1)\ln\left[\left(\prod_{i=1}^{\alpha-1} P_i\right)^{\frac{1}{\alpha-1}} \Big/ P_\alpha\right]}{\ln\left(\prod_{i=1}^{\alpha-1} i\right) - (\alpha-1)\ln\alpha} \tag{S2.2}$$

Estimating abundance

"Survey" approach

We fit a MARSS model (see S1 Text for more details) to four observation time series: two nesting time series described in S1 Text for Jamursba Medi and Wermon [9] and two abundance time series of leatherback turtles from aerial surveys off California – a central California core survey series, which includes ten data points from 1990 to 2003, and a second time series for outlying areas surveyed in only seven of those years [10]. Abundance in the survey time series is noisy, in part due to environmental variability in foraging habitat. Some of the variability in the survey abundance can be explained by the Northern Oscillation Index (NOI) [10,11], so NOI was included as a covariate affecting only the observed abundances off California (data downloaded from http://www.pfeg.noaa.gov/products/PFEL/modeled/indices/NOIx/noix_download.html on 30 April 2014). The state process model was the same as for the model used for extinction risk assessment (S1 Text), and the observation process was modeled as

$$\mathbf{y}_t = x_t + \mathbf{a} + \mathbf{D}d_t + \boldsymbol{v}_t \text{ , where } \mathbf{z}_t \sim \text{MVN}(0, \mathbf{R}) \quad \text{(S2.3)}$$

where **y** is a 4 × 22 matrix of log-transformed observations, **a** is a 4 × 1 matrix of relative scaling parameters, **d** is a 1 × 22 matrix for the NOI covariate, **D** is a 4 × 1 matrix of the effects of **d** on each set of observation (fixed to zero for all but the two lower right diagonal elements, which were modeled as equal), and **z** is a 4 × 22 matrix of observation errors with 4 × 4 covariance matrix **R**. As for the first MARSS model (S1 Text), several alternative structures for **R** were investigated, but the simplest – a model with two observation error variances, one value for the two nesting beach time series and the other for the aerial survey time series, and no covariance – had the lowest AICb value. Uncertainty distributions for parameter estimates were obtained from 2000 bootstraps of the final model. Estimates (and bootstrapped 95% confidence intervals) were: –0.058 (–0.078, –0.040) for $\hat{u}$, 0.00 (0.00, 0.004) for $\hat{q}$, −1.36 (−1.59, −1.13) for $\hat{a}_2$, −1.745 (−2.32, −1.15) for $\hat{a}_3$, –3.09 (−3.82, −2.37) for $\hat{a}_4$, 0.055 (0.022, 0.078) for the nesting beach observation variance, 0.90 (0.28, 1.36) for the foraging grounds observation variance, and 0.42 (0.08, 0.77) for the covariance of NOI and foraging grounds observations. For parameters shared between this and the extinction risk assessment model (S1 Text), estimates are almost indistinguishable between the two models.

The same process described in S1 Text was used to project the estimated state to 2014, using bootstrapped distributions for population growth rate and process variance. To predict current survey abundance estimates and uncertainty summed across both survey areas off California, the exponent of the resulting prediction was multiplied by the sum of the exponents of the bootstrapped distributions for $\hat{a}_3$ and $\hat{a}_4$ from the MARSS model, as in Equation S1.4.

References

1. Dutton DL, Dutton PH, Chaloupka M, Boulon RH. Increase of a Caribbean leatherback turtle *Dermochelys coriacea* nesting population linked to long-term nest protection. Biol Conserv. 2005;126: 186–194. doi:10.1016/j.biocon.2005.05.013

2. Eguchi T, Dutton PH, Garner SA, Alexander-Garner J. Estimating juvenile survival rates and age at first nesting of leatherback turtles at St. Croix, US Virgin Islands. In: Frick M, Panagopoulou A, Rees AF, Williams K, editors. Book of Abstracts Twenty-Sixth Annual Symposium on Sea Turtle Biology and Conservation International Sea Turtle Society, Athens, Greece. 2006. p. 292.

3. TEWG (Turtle Expert Working Group). An assessment of the leatherback turtle population in the Atlantic Ocean. 2007 p. 116.

4. Suganuma H. Leatherback turtle management of feral pig predation in Indonesia. In: Kinan I, editor. Proceedings of the Second Western Pacific Sea Turtle Cooperative Research and Management Workshop Volume I: West Pacific Leatherback and Southwest Pacific Hawksbill Sea Turtles 17-21 May 2004, Honolulu, HI Western Pacific Regional Fishery Management Council. Honolulu, HI: Western Pacific Regional Fishery Management Council; 2005. pp. 37–38.

5. Bellagio (Steering Committee, Bellagio Sea Turtle Conservation Initiative). Strategic Planning for Long-term Financing of Pacific Leatherback Conservation and Recovery: Proceedings of the Bellagio Sea Turtle Conservation Initiative, Terengganu, Malaysia; July 2007. Penang, Malaysia: The WorldFish Center; 2008 p. 79.

6. Hitipeuw C, Dutton PH, Benson S, Thebu J, Bakarbessy J. Population Status and Internesting Movement of Leatherback Turtles, *Dermochelys coriacea,* Nesting on the Northwest Coast of Papua, Indonesia. Chelonian Conserv Biol. 2007;6: 28–36. doi:10.2744/1071-8443(2007)6[28:PSAIMO]2.0.CO;2

7. Tapilatu RF, Tiwari M. Leatherback Turtle, *Dermochelys coriacea*, hatching success at Jamursba-Medi and Wermon beaches in Papua, Indonesia. Chelonian Conserv Biol. 2007;6: 154–158. doi:10.2744/1071-8443(2007)6[154:LTDCHS]2.0.CO;2

8. Caswell H. Matrix Population Models: Construction, Analysis and Interpretation. 2nd ed. Sunderland, Massachusetts: Sinauer Associates; 2001.

9. Tapilatu RF, Dutton PH, Tiwari M, Wibbels T, Ferdinandus HV, Iwanggin WG, et al. Long-term decline of the western Pacific leatherback, *Dermochelys coriacea*: a globally important sea turtle population. Ecosphere. 2013;4: art25. doi:10.1890/ES12-00348.1

10. Benson SR, Forney KA, Harvey JT, Carretta JV, Dutton PH. Abundance, distribution,and habitat of leatherback turtles (*Dermochelys coriacea*) off California, 1990-2003. Fish Bull. 2007;105: 337–347.

11. Schwing F., Murphree T, Green P. The Northern Oscillation Index (NOI): a new climate index for the northeast Pacific. Prog Oceanogr. 2002;53: 115–139. doi:10.1016/S0079-6611(02)00027-7
