# Supplementary material for: Estimating Limit Reference Points for Western Pacific Leatherback Turtles (Dermochelys coriacea) in the U.S. West Coast EEZ
Source: PLoS One. 2015 Sep 14;10(9):e0136452. doi: 10.1371/journal.pone.0136452 (PMC4569282; doi:10.1371/journal.pone.0136452)
Supplement: S3 Text — (PDF) [file pone.0136452.s003.pdf]

S3 Text. Supporting information on MSE: biological, observation, and management models

Biological model

The biological model simulated age-structured population dynamics with logistic density dependence, where the latter was generalized to a matrix model context [1], such that population growth followed $\mathbf{n}_{t+1} = \mathbf{A}_d \mathbf{n}_t$. The density-dependent transition matrix $\mathbf{A}_d$ was determined by

$$\mathbf{A}_d = \mathbf{A}_m - \left(\frac{N_K}{K}\right)^{\theta} (\mathbf{A}_m - \mathbf{A}_K) \tag{S3.1}$$

where K is carrying capacity, N_K is the population size, θ is an exponent controlling density dependent response, and $\mathbf{A}_K$ is the transition matrix at K, which was arbitrarily set to 500,000 individuals (the value of K in simulations does not affect results).

The population model was modified from [1] to include both males and females, undifferentiated, with population growth at low population sizes represented by a post-breeding-census, age-structured transition matrix $\mathbf{A}_m$ as described above (see "Estimating abundance"). For each simulated population, the "true" $\mathbf{A}_m$ was created by drawing from probability distributions (Table 2) for $\hat{\lambda}_m$, P_{adult} and P_α, and corresponding F_{adult} and F_α, and then dividing the product of the remaining survival rates by the expected value for P_{hatch} before fitting the remaining non-adult survival rates as described earlier. Populations experienced environmental stochasticity in their growth due to variation of first-year survival with each time step as a function of random draws from the probability distributions for fraction of nests destroyed by beach erosion and for hatchling emergence success (Table 2).

Density dependence was modeled with $\theta = 1$, individuals of all ages contributing equally to density dependence, and all matrix elements equally proportionally affected by density.

Consequently, $\mathbf{A}_K$ was solved numerically by reducing all matrix elements proportionally such that the eigenvalue of the matrix equalled one. Curtis and Moore [1] showed that populations with other, more plausible density dependence parameterizations resulted in similar or better management performance.

Bycatch mortality was modeled as constant with age, reflecting the assumed lack of selectivity in the CDGN for the sizes available in the California Current [2–4]. We also incorporated uncertainty in discard mortality into the biological model, with true discard mortality for each simulated population equal to a separate random draw from Beta (17.5, 8.5), based on CDGN observer data (17 deaths in 25 observed interactions) [5].

Observation model

The observation model simulates collection of data on population size. We extended the observation model described in [1] to separate systematic error in abundance estimation, e.g., due to estimated mean proportion of females, from annual estimation error, e.g., due to nest counts. An estimate of adult abundance was sampled at each time step from a log-normal distribution, with mean equal to true abundance multiplied by a scaling factor for systematic error, and coefficient of variation (CV) of 5% for annual estimation error. A 5% CV approximates the uncertainty in the projected population state, $\hat{n}_{2014}$ (S1 and S2 Texts). The scaling factor for systematic error was drawn once for each population from a probability distribution equal to the reciprocal of the product of factors in the LRP calculation that would contribute to systematic error, scaled to a mean of one. Thus, in the "naïve" approach, systematic error in abundance estimation stemmed from the factor $\frac{1}{\widehat{PF}}$, so the distribution for the scaling factor would equal that of $\widehat{PF}$ scaled to a mean of one. Systematic error for the "survey"

approach included uncertainty in the conversion factor relating nesting females at JMW to turtle abundance surveyed off California, fraction of the year spent in the WCEEZ, and parameter estimates for line-transect formulae. For the "tag" approach, systematic error included uncertainty in proportion of nesting females migrating to the WCEEZ to forage, fraction of year spent in the WCEEZ, $\widehat{PF}$, and proportion of turtles in the WCEEZ that are adults (see "Estimating abundance").

For the "naïve" approach, only nesting female abundance was observed, so total abundance was estimated within the observation model by assuming a stable age distribution (Eqn. 4). For the "survey" and "tag" approaches, relative estimation error for total abundance (and for each age class) in the observation model was the same as for the local abundance estimate, approximating the process of direct estimation of total individual turtles in the WCEEZ.

Management model

Management was simulated so that estimated annual removals from the population were equal to $\mathrm{LRP_{min}}$, i.e., a fixed lower percentile of the LRP probability distribution calculated at each time step based on the concurrent abundance estimate from the observation model. True removals varied from $\mathrm{LRP_{min}}$ due to estimation error, and fed back into the biological model in the same time step. For management models using the RVLL estimator (used with the "naïve" approach), the vector of estimated age-specific individual interactions $\hat{\mathbf{i}}$ at each time step was calculated from estimated discard mortality and stage-specific mortality, weighted by abundance and estimated reproductive value. Thus $\hat{\mathbf{i}} = \frac{RVLL_{\min}}{\hat{m}_{\mathrm{discard}}} \mathbf{u}_r \mathbf{n}_t / \sum(\mathbf{u}_r \mathbf{n}_t \hat{\mathbf{v}}_m)$, where $\hat{m}_{\mathrm{discard}}$ is estimated discard mortality (the point estimate, 0.68) [5] and $\mathbf{u}_r$ is relative age-specific bycatch

rates. For this study, we assumed that the number of interactions (deaths plus animals released alive) was measured without error (i.e., 100% observer coverage), because the resulting LRP estimates are so low (see Results) that for corresponding removals, estimation error from an observer program with incomplete coverage would change year to year with number of interactions and thus also secularly with population abundance [6]. Thus, modeled estimation error in removals derived solely from uncertainty in discard mortality. True age-specific removals equalled $m_{\text{discard}} \frac{\hat{\mathbf{\imath}}}{\sum \hat{\mathbf{\imath}}}$, where m_{discard} is true discard mortality. For management models using the modified PBR estimator ("survey" and "tag" approaches), calculations were the same as for the naïve approach except estimated age-specific interactions were calculated as $\hat{\mathbf{\imath}} = \frac{LRP_{\min}}{\hat{m}_{\text{discard}}} \mathbf{u}_r \mathbf{n}_t / \sum(\mathbf{u}_r \mathbf{n}_t)$. True age-specific removals were then fed back into the biological model.

References

1. Curtis KA, Moore JE. Calculating reference points for anthropogenic mortality of marine turtles. Aquat Conserv Mar Freshw Ecosyst. 2013;23: 441–459. doi:10.1002/aqc.2308

2. Eckert SA. Distribution of juvenile leatherback sea turtle Dermochelys coriacea sightings. Mar Ecol Prog Ser. 2002;230: 289–293. doi:10.3354/meps230289

3. Benson SR, Eguchi T, Foley DG, Forney KA, Bailey H, Hitipeuw C, et al. Large-scale movements and high-use areas of western Pacific leatherback turtles, *Dermochelys coriacea*. Ecosphere. 2011;2: art84. doi:10.1890/ES11-00053.1

4. Jones TT, Hastings MD, Bostrom BL, Pauly D, Jones DR. Growth of captive leatherback turtles, *Dermochelys coriacea*, with inferences on growth in the wild: Implications for population decline and recovery. J Exp Mar Biol Ecol. 2011;399: 84–92. doi:10.1016/j.jembe.2011.01.007

5. NMFS (National Marine Fisheries Service). Biological opinion on the continued management of the drift gillnet fishery under the Fishery Management Plan for U.S. West Coast Fisheries for Highly Migratory Species 2012/03020:DDL. Long Beach, CA: National Marine Fisheries Service, Southwest Region; 2013.

6. Martin SL, Stohs SM, Moore JE. Bayesian inference and assessment for rare-event bycatch in marine fisheries: a drift gillnet fishery case study. Ecol Appl. 2015;25: 416–429. doi:10.1890/14-0059.1
