# Supplementary material for: Estimating Limit Reference Points for Western Pacific Leatherback Turtles (Dermochelys coriacea) in the U.S. West Coast EEZ
Source: PLoS One. 2015 Sep 14;10(9):e0136452. doi: 10.1371/journal.pone.0136452 (PMC4569282; doi:10.1371/journal.pone.0136452)
Supplement: S4 Text — (PDF) [file pone.0136452.s004.pdf]

S4 Text. Supporting information on sensitivity trial methods

Underestimation of age at first reproduction

In order to allow estimated and true age at first reproduction ($\hat{\alpha}$ and α, respectively) to be different, we needed to convert between estimated age-classified vectors and true age-classified vectors of different length

First, we needed to convert true age-classified population size from an α+1 to an $\hat{\alpha} + 1$ vector for estimation. When the age distribution of the population was unknown, we simply extrapolated age-classified abundance from sampled adult abundance based on the estimated transition matrix for the population. If age distribution was known, we converted true age distribution to the estimated number of age classes by dividing each age class except adults into $\hat{\alpha}$ equal fractions, then summing α of the resulting proportional abundances at a time to obtain relative abundance in $\hat{\alpha}$ juvenile and sub-adult classes. Adult abundance remained the same. Estimated abundance was then sampled from true abundance as described originally for known age distribution.

Second, after estimating the limit reference point, we needed to convert estimated interactions from an $\hat{\alpha} + 1$ vector to an α+1 vector, so we could calculate true age-classified interactions and removals and pass these back to the biological model. We divided abundance in each estimated age class into α+1 fractions, weighted by true relative abundance in the corresponding true age classes (true abundance in the destination age class divided by the abundance in the corresponding age class of true abundance converted to estimated abundance), and summed $\hat{\alpha}$ of the resulting fractional abundances at a time to obtain the true age distribution of interactions.

Steeper initial increase in juvenile survival rates

To fit mean juvenile survival to a steeper increase than that used for both estimated and true transitions matrices in the base trials, we specified a shallow linear trend for survival in the last $\alpha/2$ stages (rounded to a whole number), factored out the resulting survival rates from the product of all juvenile survival rates, and fit the curve used for estimating all juvenile survival rates to the remaining juvenile age classes. The linear trend specified for the later juvenile age classes was $P_i = 0.05\ i/\mathrm{round}(\alpha/2) + P_\alpha - 0.05\ \alpha/\mathrm{round}(\alpha/2)$.

Unstable starting age distribution

A "pessimistic" (high ratio of adults to other age classes) starting age distribution for each population was determined by simulating the effect of historic high egg mortality due to harvests in the early 1980s [1], and ongoing high mortality of juvenile and adult stages on the age distribution of each of 2000 simulated populations, given its unique transition matrix. Each population was initialized at stable age distribution at *K*, and then subjected to a 95% reduction in fertility for 14 years, followed by an ongoing 20% reduction for the remaining 26 years; meanwhile, survival rates for the last 6 age/stage classes, including adults, were decreased such that the eigenvalue of the estimated transition matrix subject to those decreases would match the observed population growth rate of 0.94 [1], with adult survivorship diminished 33% more than the other affected stages. The result was a reduction in survival rates of 0.15 for late juvenile stages and 0.2 for adults, which was applied over the full 40 years of the simulation. The age distribution with the highest ratio of adult abundance to total adult equivalents in the population (based on the estimated transition matrix) for each simulated population in the last ten years of

the simulation was used to initialize the corresponding population's trajectory in the sensitivity trial.

References

1. Tapilatu RF, Dutton PH, Tiwari M, Wibbels T, Ferdinandus HV, Iwanggin WG, et al. Long-term decline of the western Pacific leatherback, *Dermochelys coriacea*: a globally important sea turtle population. Ecosphere. 2013;4: art25. doi:10.1890/ES12-00348.1
