# Supplementary material for: Estimating Limit Reference Points for Western Pacific Leatherback Turtles (Dermochelys coriacea) in the U.S. West Coast EEZ
Source: PLoS One. 2015 Sep 14;10(9):e0136452. doi: 10.1371/journal.pone.0136452 (PMC4569282; doi:10.1371/journal.pone.0136452)
Supplement: S5 Text — (PDF) [file pone.0136452.s005.pdf]

S5 Text. R functions for MSE

R Function #1: PopGrow.lmin

```
PopGrow.lmin = function(nsim, tsim, dem.est, dem, kstg, ddstr,
    theta=1, es=list(flag=FALSE), cvn.est, n.sys=1, pd.survey=1,
    cvm.est, mdisc.est, mdisc, Nbias=1, mbias=1, cvn.bias=1,
    cvm.bias=1, b=0.5, lam.lrp=NA, p.lmin, fa=1, n0, K,
    agedis.start="stableMax", agedis.t0.user=NA, rbr=NA,
    lrp.ind=FALSE, stdis.info="naive") {

  # function projects a population subject to bycatch mortality
    determined by LRP applied at a given percentile value
    (p.lmin)
  # Notes:
  # - designed for post-breeding census matrix, so there are
    afr+1 age classes, and environmental stochasticity in
    transition rates acts on fertilities too
  # - Most easily applied via SimPopGrowth.LRP wrapper function
  # - The number of values in each distribution input for lrp
    equals nsim

  # INPUT:
  # nsim = number of simulations to run with given parameters to
    get distribution of results
  # tsim = c(years before bycatch and management start
    occurring, total time length of simulation)
    # Note: To simulate population growth without bycatch, set
    tsim = c(number of years to simulate without bycatch, total
    number of years in simulation).
  # dem.est = list of estimated life history parameters at zero
    abundance:
    # (a) stgdur = vector of stage durations
    # (b) trans = vector of stage-specific transition rates (aka
    survival, as used here)
    # (c) f = vector of fertilities (offspring per adult)
      # Fertility must be provided per age/matrix element (not
    per stage as for trans). Can omit leading zeros.
  # dem = list of "true" life history parameters at zero
    abundance:
    # (a) stgdur = vector of stage durations
    # (b) trans = matrix (nstg* 1 or nsim) with "true" values
    Pad, Pj (if using fixed values without uncertainty, send as
    matrix with one column)
    # (c) f = matrix (1 or nsim columns, must match ncol of
    trans) of (mean, in case of environmental stochasticity) true
    fertilities
```

```
    # Fertility must be provided per age/matrix element (not
  per stage as for trans). Can omit leading zeros.
# kstg = logical vector indicating which stages contribute to
  carrying capacity
# ddstr = vector of relative strength of density dependence on
  each matrix element (currently trans and fert; zero if not
  density dependent)
  # Note that post-breeding census, so ddstr for fertilities
  must be at least that of corresponding transition rates, and
  stronger if want fecundity to be density-dep
# theta = exponent controlling density dependent response
# es = list specifying presence and parameters of
  environmental stochasticity in population growth
  # (1) flag = logical scalar indicating whether growth is
  stochastic (TRUE) or deterministic (FALSE)
  # (2) t.scale (optional) = array
  (dim(dem$trans)[1]*tsim[2]*nsim) of scaling factors
  determining environmental stochasticity of transition rates
  # (3) f.scale (optional) = array (dim(dem$f)[1] OR
  1*tsim[2]*nsim) of scaling factors determining environmental
  stochasticity of fertilities
  # Note that fertilities will be modulated by corresponding
  t.scale, so only include variability in egg production in
  f.scale
# cvn.est = scalar; estimated CV for random uncertainty in
  population abundance estimate
# n.sys = vector of systematic sampling biases for population
  (e.g. due to estimated proportion female (true/est), RI,
  clutch frequency)
# pd.survey = integer scalar; periodicity of population
  surveys
# cvm.est = numeric scalar; estimated CV of bycatch mortality
  estimate
# mdisc.est = numeric scalar between 0 and 1; estimated
  discard mortality
# mdisc = vector with sampled true discard mortality for each
  sim
# Nbias = bias in population abundance estimation
  (expected/actual abundance)
# mbias = bias in mortality estimation (expected/actual
  mortalities)
# cvn.bias = bias in CV estimation for abundance
  (estimated/true CVn; Note: bias in CV estimation of abundance
  affects validity of Lmin estimation)
# cvm.bias = bias in CV estimation for mortality
  (estimated/true CVm; Note: bias in CV estimation of mortality
  affects performance of original "optimum" p.lmin choice)
```

```
# b = numeric vector; multiplier(s) of lambda in MDG equation,
  specific to density dependence type and stochasticity of
  lambda
# lam.lrp = scalar or vector of lambda(s) to use when
  calculating limit reference point (distribution). If missing,
  will use scalar lambda corresponding to dem.est vital rates
# p.lmin = numeric scalar; percentile at which LRP performance
  evaluated
# fa = numeric scalar; recovery factor which acts as
  multiplier of lambda in LRP equation
# n0 = scalar or vector(nsim) of population abundance in
  stages contributing to proportion of carrying capacity
# K = carrying capacity for stages contributing to it
# agedis.start =  choice of age distribution at beginning of
  simulations; options: "stableMax" (default; uses stable stage
  dist for A at zero population), "stableDD" (uses stable stage
  distribution for Ad at start population size), and "userDef"
# agedis.t0.user = matrix (afr+1 * 1 or nsim) with user-
  defined starting age distribution(s)
# rbr = relative age-specific bycatch rates
# lrp.ind = logical scalar; TRUE means bycatch is managed
  based on number of individuals (PBR), not rv-weighted
  population (RVLL)
# stdis.info has options "naive" (assume stable stage
  distribution for population) and "known" (only implemented
  for dem and dem.est with same number of stages and stage
  durations)

#OUTPUT:
# outcomes for a population managed according to RV-weighted
  LRP (aka RVLL) or individual-based LRP (aka PBR) (latter if
  lrp.ind=TRUE)
# (a) ntot = matrix of total number of individuals in
  population (tsim*nsim)
# (b) ad = matrix of number of adults (tsim*nsim)
# (c) ad.est = matrix of estimated number of adults
  (tsim*nsim)
# (c) n.k = matrix of proportion of carrying capacity
  (tsim*nsim)
# (d) lmin = matrix of lmin (equal to estim take, in AE or
  individuals) (tsim*nsim)
# (e) ntot.mort = matrix of true total mortalities (numbers)
  (tsim*nsim)

# QA/QC
if (tsim[2]>tsim[1] && is.na(rbr[1])) stop("If bycatch occurs
  in some or all years, must supply rbr.")
```

```
afr = sum(dem$stgdur)-1  # age at first reproduction

if (es$flag) {
  if (invalid(es$t.scale)) { es$t.scale = array(1,
  c(dim(dem$trans)[1], tsim[2], nsim))
  } else {
    if (dim(es$t.scale)[2]!=tsim[2]) stop("Number of columns
  in es$t.scale must equal tsim[2].")
    if (dim(es$t.scale)[3]!=nsim) stop("Array depth of
  es$t.scale must equal nsim.")
    if (dim(es$t.scale)[1]!=dim(dem$trans)[1]) stop("Number of
  rows in es$t.scale must equal number of stage-specific
  transition rates specified (dim(dem$trans)[1]).")
    if (any(es$t.scale < 0)) stop("No negative values allowed
  in es$t.scale.")
    if (any(es$t.scale * aperm(array(dem$trans,
  c(dim(dem$trans)[1],nsim,tsim[2])), c(1,3,2)) > 1)) stop("No
  values allowed in es$t.scale such that resulting transition
  rate is greater than 1.")
  }
  nf = dim(dem$f)[1]  # number of stages with fertility values
  provided
  sfi = c(rep(FALSE, afr+1-nf), rep(TRUE,nf)) # index
  transition rates that correspond to positive fertility values
  if (invalid(es$f.scale)) { es$f.scale = array(1, c(nf,
  tsim[2], nsim))
  } else {
    if (dim(es$f.scale)[2]!=tsim[2]) stop("Number of columns
  in es$f.scale must equal tsim[2].")
    if (dim(es$f.scale)[3]!=nsim) stop("Array depth of
  es$f.scale must equal nsim.")
    if (dim(es$f.scale)[1]==1) es$f.scale = array(
  rep(es$f.scale,each=nf), c(nf, tsim[2], nsim)) # if only one
  row, repeat for dim(dem$f)[1]
    if (any(es$f.scale < 0)) stop("No negative values allowed
  in es$f.scale.")
  }
}

# get estim max population growth rate, stable stage
  distribution, and RVs from estimated transition matrix
A.est = mkPopMatrix(dem.est$stgdur, dem.est$trans, dem.est$f)
  # estimated transition matrix at zero abundance
eig.est = eigen.analysis(A.est)
lam.est = eig.est$lambda1
rv.est = eig.est$repro.value
```

```
stst.est = eig.est$stable.stage
afr.est = sum(dem.est$stgdur)-1
# Default lambda to be used in LRP estimation corresponds to
  dem.est
if (is.na(lam.lrp[1])) lam.lrp = lam.est

dem.k = findVRmod(dem, lam.mod=1, ddstr, method="root")  #
  derive true life history parameters at carrying capacity
vr = rbind(dem$trans, dem$f)  # matrix of true vital rates,
  including survival rates and fertility
vrk = rbind(dem.k$trans, dem.k$f)
# create logical index to age/stage classes contributing to
  carrying capacity (length equal to sum of dem$stgdur -1)
ki = indexDDstages(kstg=kstg, dem$stgdur)

# Initialize objects
N = array(NA,dim=c(afr+1,tsim[2]+1,nsim))  # age-specific N
  (rows) through time (columns) over nsim
N.est = AE.est = array(NA,dim=c(afr.est+1,tsim[2],nsim)) #
  estimated abundance in terms of individuals and adult
  equivalents
n.k = matrix(NA,tsim[2]+1,nsim) # percent carrying capacity
aetot.est = matrix(NA,tsim[2],nsim)  # total estimated adult
  equivalents through time
lmin = ixn.est = ntot.mort = ntot.ixn =
  matrix(NA,tsim[2],nsim)  # lmin (equal to total estimated
  mortality in adult equivalents), estimated fishery
  interactions (in ae or n), total byc mort and interactions in
  terms of individuals
N.mort = array(NA,dim=c(afr+1,tsim[2],nsim))  # true age-
  specific (rows) bycatch mortality through time (columns) over
  nsim
N.ixn.est = array(NA,dim=c(afr.est+1,tsim[2],nsim))  #
  estimated age-specific bycatch interactions

# derived parameters and bookkeeping shortcuts
sigma.n.est = sqrt(log((cvn.est/cvn.bias)^2+1))  # get true
  estimation sigma for log-transformed, log-normal censused
  portion of population (nesting adult females in current
  formulation)
sigma.n.lrp = sqrt(log(cvn.est^2+1))  # use estimated cvn.est
  to estimate uncertainty around population going into LRP
  distribution
if(tsim[1]<tsim[2]) sigma.mort =
  sqrt(log((cvm.est/cvm.bias)^2+1))  # get sigma for log-
  transformed, log-normal mortality/interaction distribution
```

```
y.byc = c(rep(FALSE,tsim[1]),rep(TRUE,diff(tsim)))  # vector
  of length tsim[2] indicating for each year whether bycatch
  and management occur

# Initial conditions
# derive starting age distribution
agedis.t0 = set.agedis(type=agedis.start, n0, K, dem, dem.k,
  theta, agedis.t0.user)
# initialize population abundance by prorating n0 to stages
  contributing to K, with abundance in all stages distributed
  according to agedis.t0
N[,1,] = matrix(n0,afr+1,nsim,byrow=TRUE) *
  matrix(agedis.t0,afr+1,nsim)

# project N for tsim time steps
for (ti in 1:tsim[2]) {

  # project true population growth
  n.k[ti,] = colSums(N[ki,ti,])/K  # calculate percent
  carrying capacity occupied
  vrd = matrix(vr,dim(vr)[1],nsim) -
  matrix((n.k[ti,]^theta),dim(vr)[1],nsim,byrow=TRUE) *
  matrix(vr - vrk,dim(vr)[1],nsim)  # calculate density-
  adjusted vital rates
  vrd = matrix(pmax(0.0001, vrd),dim(vr)[1],nsim)  # safeguard
  against negative or zero vital rates

  for (nsi in 1:nsim) {
    if (es$flag) {
      Ad = mkPopMatrix(dem$stgdur,
  vrd[1:dim(dem$trans)[1],nsi]*es$t.scale[,ti,nsi],
  vrd[(dim(dem$trans)[1]+1):dim(vr)[1],nsi]*es$f.scale[,ti,nsi]
  *es$t.scale[sfi,ti,nsi])
    } else {
      Ad = mkPopMatrix(dem$stgdur,
  vrd[1:dim(dem$trans)[1],nsi],
  vrd[(dim(dem$trans)[1]+1):dim(vr)[1],nsi])
    }
    N[,ti+1,nsi] = Ad %*% N[,ti,nsi]
  }
  N.afrest =
  rbind(apply(array(rep(N[1:(afr),ti+1,],each=afr.est)/(afr.est
  ),c(afr,afr.est,nsim)),c(2,3),sum),N[afr+1,ti+1,])

  # sample population and estimate abundances and AE
  if ((ti %% pd.survey==1)|(pd.survey==1)) {  # check whether
  this is a survey year
```

```
  mu.n.est = log(N[afr+1,ti+1,]*Nbias*n.sys) -
0.5*sigma.n.est^2  # get mu for log-transformed, log-normal
adult population distribution, including systematic
uncertainties
  nafr.est = rlnorm(nsim, meanlog=mu.n.est,
sdlog=sigma.n.est)  # sample adult population size with
(biased) expected value = N[afr+1]*Nbias*n.sys and true CV =
cvn.est/cvn.bias
  N.est[,ti,] = switch(stdis.info,  # estimate stage-
distributed abundance
                       naive =
matrix(nafr.est,afr.est+1,nsim,byrow=TRUE)*(stst.est/stst.est
[afr.est+1]),   # based on a stable-stage distribution
assumption
                       known =
matrix(nafr.est/N[afr+1,ti+1,],afr.est+1,nsim,byrow=TRUE)*N.a
frest)  # based on perfect knowledge of true stage
distribution
  AE.est[,ti,] = N.est[,ti,] * (rv.est/rv.est[afr.est+1])  #
convert to stage-distributed, RV-weighted abundance
} else {
  N.est[,ti,] = N.est[,ti-1,]
  AE.est[,ti,] = AE.est[,ti-1,]
}
aetot.est[ti,] = colSums(AE.est[,ti,])  # estimate of total
adult equivalents

if (y.byc[ti]) {
  # calculate LRP distribution and Lmin for management
  if (!lrp.ind) { mu.n.lrp = log(aetot.est[ti,])  # use last
population estimate in terms of AE for LRP calculation
  } else { mu.n.lrp = log(colSums(N.est[,ti,])) } # if
managing based on individuals,  use population in terms of
individuals to get lmin
  lrp = b * (lam.lrp-1) * (1/n.sys) * matrix(rlnorm(nsim^2,
meanlog=mu.n.lrp, sdlog=sigma.n.lrp),nsim,nsim,byrow=TRUE) *
fa  # calculate estimated distributions for LRP for each
simulation
  lmin[ti,] = apply(lrp,sort,MARGIN=2)[round(p.lmin*nsim),]
# sort distributions for each simulation and index at p.lmin
  ixn.est[ti,] = lmin[ti,]/mdisc.est  # calculate total
estimated interactions in terms of individuals or RV

  # convert estimated total bycatch interactions (in RV or
indiv) to estimated age-classified individual interactions
  if (!lrp.ind) {   # bycatch mortality managed based on rv-
weighted population
```

```
    # estimated age-specific AE intrxns proportional to age-
specific bycatch rates * true age-spec pop * RVs used by mgt
to calc intrxns in terms of AEs
    denom.ae = colSums(rbr * N.afrest * rv.est)
    AE.ixn.est = matrix(NA, afr.est+1, nsim)
    AE.ixn.est[,denom.ae>0] =
matrix(ixn.est[ti,denom.ae>0],afr.est+1,sum(denom.ae>0),byrow
=TRUE) * (rbr * N.afrest[,denom.ae>0] *
rv.est)/matrix(denom.ae[denom.ae>0],afr.est+1,sum(denom.ae>0)
,byrow=TRUE)  # estim age-specific morts in AEs (we know rbr)
    AE.ixn.est[,denom.ae==0]=0
    N.ixn.est[,ti,] = AE.ixn.est /
(rv.est/rv.est[afr.est+1])  # estim age-specific intrxns
  } else {  # bycatch interactions managed based on
individuals
    denom = colSums(rbr * N.afrest)
    N.ixn.est[,ti,denom>0] =
matrix(ixn.est[ti,denom>0],afr.est+1,sum(denom>0),byrow=TRUE)
* (rbr *
N.afrest[,denom>0])/matrix(denom[denom>0],afr.est+1,sum(denom
>0),byrow=TRUE)
    N.ixn.est[,ti,denom==0]=0
  }

  # sample actual bycatch interactions with true expected
value = nixn.est[afr+1]/mbias and true CV = cvm.est/cvm.bias
  mu.ixn = log(colSums(N.ixn.est[,ti,])/mbias) -
0.5*sigma.mort^2  # get mu for log-transformed, log-normal
interactions distribution
  ntot.ixn[ti,] = rlnorm(nsim, meanlog=mu.ixn,
sdlog=sigma.mort)  # sample true adult bycatch interactions
  ntot.mort[ti,] = ntot.ixn[ti,]*mdisc
  # convert age distribution for estimated interactions to
(potentially different number of stages for) age distribution
of true mortality
  N.mort[,ti,] = 0
  imort = ntot.mort[ti,]>0
  num1 =
array(rep(N.ixn.est[,ti,imort],each=afr+1),c(afr.est+1,afr+1,
sum(imort)))
  num2 =
array(rep(N[,ti+1,imort],each=afr.est+1),c(afr.est+1,afr+1,su
m(imort)))
  temp1 =
array(rep(N[,ti+1,imort],each=afr.est+1),c(afr+1,afr.est+1,su
m(imort)))
  temp2 = apply(temp1,c(2,3),sum)
```

```
      denom =
    array(rep(temp2,each=afr+1),c(afr.est+1,afr+1,sum(imort)))
      izero = denom==0
      temp3 = array(NA,c(afr.est+1,afr+1,sum(imort)))
      temp3[!izero] = num1[!izero]*num2[!izero]/denom[!izero]
      temp3[izero] = 0
      temp4 = apply(temp3,c(2,3),sum)
      agedis.mort =
    temp4/matrix(colSums(temp4),afr+1,sum(imort),byrow=TRUE)
      rm(temp1,temp2,temp3,temp4,izero)
      N.mort[,ti,imort] =
    matrix(ntot.mort[ti,imort],afr+1,sum(imort),byrow=TRUE)*agedi
    s.mort   # true individual mortalities (currently have same
    stage distribution as estimated)
    } else {
      lmin[ti,] = ixn.est[ti,] = ntot.mort[ti,] = ntot.ixn[ti,]
    = 0
      N.mort[,ti,] = 0
      N.ixn.est[,ti,] = 0
    }

    # apply bycatch mortality to population
    N[,ti+1,] = pmax(0, N[,ti+1,] - N.mort[,ti,])
  }

  n.k[ti+1,] = colSums(N[ki,ti+1,])/K  # calculate final percent
    carrying capacity occupied

  return(list(ntot=apply(N,c(2,3),sum), ad=N[afr+1,,],
    ad.est=N.est[afr.est+1,,], n.k=n.k, lmin=lmin,
    ntot.mort=ntot.mort))
}
```

R Function #2: findVRmod

```
findVRmod = function(dem.init, lam.mod, vr.wts, method="optim")
    {

  # findVRmod finds the corresponding vital rates for a given
    population growth rate (lam.mod),
  # based on the original vital rates, stage durations, and
    relative weights controlling how much each vital rate changes

  # ARGUMENTS:
```

```
# dem.init = list of life history parameters in format used
  for dem elsewhere:
  # (a) stgdur = vector of stage durations
  # (b) trans = matrix (length(stgdur)* 1 or nsim) with Pi (if
  using fixed values without uncertainty, send as matrix with
  one column)
  # (c) f = matrix (1 or nsim columns, must match ncol of
  trans) of (mean) true fertilities
    # Fertility must be provided per age/matrix element (not
  per stage as for trans). Can omit leading zeros.
# lam.mod = scalar; lambda of output transition matrix. Must
  be less than lambda of input transition matrix.
# vr.wts = relative change of each vital rate between input
  and output. Must include one for each trans and f.
# method = "optim" to use optimizer to find values such that
  lambda=lam.mod, or "root" for root-finder (CAUTION:
  implemented for age-based matrix only)
  # Note: "root" is the faster method

#OUTPUT:
# list with modified life history parameters, components of
  transition matrix with eigenvalue lam.mod
  # (a) stgdur = vector of stage durations
  # (b) trans = vector of stage-specific transition (aka
  survival, here) rates
  # (c) f = scalar or vector (nsim) of mean fertility

# initialize matrix of new vital rates
nvr = length(dem.init$stgdur)+dim(dem.init$f)[1]
if (length(vr.wts) != nvr) stop("Length of vr.wts must equal
  number of unique vital rates (trans and f) defined in
  dem.init.")
p = rep(NA,dim(dem.init$f)[2])
si= 1:length(dem.init$stgdur)
fi = (nvr-dim(dem.init$f)[1]+1):nvr

if(method=="optim") {  # solve through optimization
  lamdiff <- function(prop,vr,vr.wts) {
    vr.mod = vr * (1 - prop * vr.wts)
    A.temp = mkPopMatrix(dem.init$stgdur, vr.mod[si],
  vr.mod[fi])
    lam = lambda(A.temp)
    abs(lam - lam.mod)
  }
  for (i in 1:dim(dem.init$f)[2])
```

```
      p[i] =
    optimize(f=lamdiff,interval=c(0,1),vr=c(dem.init$trans[,i],de
    m.init$f[,i]),vr.wts=vr.wts)$minimum
  } else if (method=="root") {    # solve by finding root of
    characteristic equation for modified lambda
    for (i in 1:dim(dem.init$f)[2]) {
      nm = sum(dem.init$stgdur)  # size of transition matrix
      nf = dim(dem.init$f)[1]  # number of stages with fertility
    values provided
      f = c(rep(0, nm-nf), dem.init$f[,i])  # fertility vector
      s = rep(dem.init$trans[,i],dem.init$stgdur)  # survival
    vector
      vrwf = c(rep(0, nm-nf),
    vr.wts[(dim(dem.init$trans)[1]+1):length(vr.wts)])  #
    relative dd weights for fertility
      vrws =
    rep(vr.wts[1:dim(dem.init$trans)[1]],dem.init$stgdur)  #
    relative dd weights for survival
      p[i] = uniroot( function(x) { csum = 0; for(ci in 1:nm)
    csum = csum +
        lam.mod^(-1*ci) * ((1-x*vrwf[ci])*f[ci] * prod((1-
    x*vrws[0:(ci-1)])*s[0:(ci-1)]) +
        (1-x*vrws[nm])*s[nm] * prod(-1*(1-x*vrwf[(ci-1):(ci-
    1)])*f[(ci-1):(ci-1)]) * prod((1-x*vrws[0:max(0,ci-
    2)])*s[0:max(0,ci-2)]));
        csum = csum - 1 },
        c(0,1))$root
    }
  }
  vr.mod = rbind(dem.init$trans,dem.init$f) * (1 - vr.wts %*%
    matrix(p,nrow=1))
  return(list(stgdur=dem.init$stgdur,
    trans=as.matrix(vr.mod[si,]), f=as.matrix(vr.mod[fi,])))
}
```

R Function #3: getbetascales

```
getbetascales = function(x){

  # get beta scale values for given mean and standard deviation
  # x is input vector of values in distribution to be
    characterized by beta distribution

  m = mean(x)
  s2 = var(x)
```

```
  a = ((1-m)/s2 - 1/m)*m^2
  b = a*(1/m - 1)

  return(c(a,b))
}
```

R Function #4: indexDDstages

```
indexDDstages = function(kstg, stgdur) {
  # INPUT:
  # kstg = logical vector specifying which stages contribute to
    carrying capacity
  # stgdur = integer vector specifying durations of each stage

  # OUTPUT:
  # ki = index to age/stage classes that contribute to carrying
    capacity
      # logical vector with length equal to dimension of
    population transition matrix

  # function indexes which age classes (based on stages and
    stage durations) contribute to carrying capacity
  # and relative decreases in stage-specific vital rates as
    population increases

  nstages = length(stgdur)

  # index stages whose abundance contributes to fraction of
    carrying capacity
  ki = logical(0)
  for (i in 1:nstages) {
    ki = c(ki,rep(kstg[i],stgdur[i]))
  }

  return(ki)
}
```

R Function #5: set.agedis

```
set.agedis = function(type="stableMax", n0, K, dem, dem.k,
    theta=1, agedis.user=NA) {
  # Input:
```

```
  # type = choice of age distribution at beginning of
    simulations
    # options:
      # "stableMax" (default; uses stable stage distribution for
    A at zero population)
      # "stableDD" (uses stable stage distribution for Ad at
    start population)
      # "userDef"  (user-defined starting age distribution)
  # n0 = scalar or vector(nsim) of population abundance in
    stages contributing to proportion of carrying capacity
  # K = carrying capacity for stages contributing to it
  # dem = true mean vital rates at abundance zero
  # dem.k = true mean vital rates at carrying capacity
  # theta = exponent controlling density dependent response
  # agedis.user = matrix with user-provided stage distribution

  # Output:
  # age distribution of starting population for simulations (sum
    equals one)

  agedis = matrix(NA,nrow=sum(dem$stgdur),ncol=dim(dem$f)[2])
  n0 = matrix(n0,1,dim(dem$f)[2])

  if (type=="stableMax") {
    for (i in 1:dim(dem$f)[2]) {
      A = mkPopMatrix(dem$stgdur,dem$trans[,i],dem$f[,i])
      agedis[,i] = stable.stage(A)
    }
  } else if (type=="stableDD") {
    for (i in 1:dim(dem$f)[2]) {
      n.k = n0[i]/K
      A = mkPopMatrix(dem$stgdur,dem$trans[,i],dem$f[,i])
      Ak = mkPopMatrix(dem.k$stgdur,dem.k$trans[,i],dem.k$f[,i])
      Ad = A - (n.k^theta)*(A - Ak)
      Ad[Ad<0] = 0.0001  # safeguard against negative or zero
    fertility
      agedis[,i] = stable.stage(Ad)
    }
  } else if (type=="userDef") {   # standardize so sum to 1
    agedis =
    agedis.user/matrix(colSums(agedis.user),nrow=sum(dem$stgdur),
    ncol=dim(dem$f)[2],byrow=TRUE)
  }

  return(agedis)
}
```

R Function #6: mkPopMatrix

```
mkPopMatrix = function(stgdur, trans, fertility) {
  # function creates an age-classified transition matrix with
    adults treated as a single stage

  # ARGUMENTS:
  # stgdur = number of years in each corresponding stage class
    (final adult class must have stage duration of 1)
  # trans = vector of stage-specific transition probabilities to
    the next age (i.e., survivals)
  # fertility = offspring per individual of each age/stage (min
    length of one corresponding to final adult stage class).
    # Must be provided per age/matrix element (not per stage as
    for trans). Can omit leading zeros.

   nm = sum(stgdur)  # size of transition matrix
   A = matrix(0,nm,nm)
   s = rep(trans,stgdur)
   A[row(A)==col(A)+1] = s[1:(nm-1)]  # assign survival rates to
    the matrix subdiagonal
   A[nm,nm] = s[nm]  # assign survival rates for final adult
    stage
   A[1,(nm-(length(fertility)-1)):nm] = fertility
   A
}
```
